# Development and Validation of CRISPR Activator Systems for Overexpression of CB1 Receptors in Neurons

**DOI:** 10.3389/fnmol.2020.00168

**Published:** 2020-09-08

**Authors:** Valentina Di Maria, Marine Moindrot, Martin Ryde, Antonino Bono, Luis Quintino, Marco Ledri

**Affiliations:** ^1^Laboratory of Molecular Neurophysiology and Epilepsy, Department of Clinical Sciences, Epilepsy Center, Lund University, Lund, Sweden; ^2^Laboratory of CNS Gene Therapy, Department of Experimental Medical Sciences, Lund University, Lund, Sweden

**Keywords:** CRISPR, CB1, Cas9 activators, AAV (Adeno-Associated virus), lentivirus, gene therapy

## Abstract

Gene therapy approaches using viral vectors for the overexpression of target genes have been for several years the focus of gene therapy research against neurological disorders. These approaches deliver robust expression of therapeutic genes, but are typically limited to the delivery of single genes and often do not manipulate the expression of the endogenous locus. In the last years, the advent of CRISPR-Cas9 technologies have revolutionized many areas of scientific research by providing novel tools that allow simple and efficient manipulation of endogenous genes. One of the applications of CRISPR-Cas9, termed CRISPRa, based on the use of a nuclease-null Cas9 protein (dCas9) fused to transcriptional activators, enables quick and efficient increase in target endogenous gene expression. CRISPRa approaches are varied, and different alternatives exist with regards to the type of Cas9 protein and transcriptional activator used. Several of these approaches have been successfully used in neurons *in vitro* and *in vivo*, but have not been so far extensively applied for the overexpression of genes involved in synaptic transmission. Here we describe the development and application of two different CRISPRa systems, based on single or dual Lentiviral and Adeno-Associated viral vectors and VP64 or VPR transcriptional activators, and demonstrate their efficiency in increasing mRNA and protein expression of the *Cnr1* gene, coding for neuronal CB1 receptors. Both approaches were similarly efficient in primary neuronal cultures, and achieved a 2–5-fold increase in *Cnr1* expression, but the AAV-based approach was more efficient *in vivo.* Our dual AAV-based VPR system in particular, based on *Staphylococcus aureus* dCas9, when injected in the hippocampus, displayed almost complete simultaneous expression of both vectors, high levels of dCas9 expression, and good efficiency in increasing *Cnr1* mRNA as measured by *in situ* hybridization. In addition, we also show significant upregulation of CB1 receptor protein *in vivo*, which is reflected by an increased ability in reducing neurotransmitter release, as measured by electrophysiology. Our results show that CRISPRa techniques could be successfully used in neurons to target overexpression of genes involved in synaptic transmission, and can potentially represent a next-generation gene therapy approach against neurological disorders.

## Introduction

Gene therapy approaches using engineered viral vectors have been for many years the focus of developing alternative treatment strategies against several neurological disorders, such as Parkinson’s disease, Alzheimer’s Disease and epilepsy. These strategies rely on the delivery of therapeutic genes in specific areas of interest and aim to interfere with disease processes or restore pathological alterations in brain networks key to the development of disease symptoms (Simonato et al., 2013; Simonato, 2014; Combs et al., 2016; Axelsen and Woldbye, 2018; Ingusci et al., 2019). Similar approaches, including also the use of transgenic animals or knock-down approaches, have also been used extensively to interrogate the function of specific genes in particular disease states. While valuable, these techniques often do not manipulate the expression of the endogenous gene locus and are mostly limited to affecting one target gene at a time (Kunieda et al., 2002; Götz and Ittner, 2008; Harvey et al., 2008, 2012; Dawson et al., 2018).

In the last years, CRISPR-Cas9 (clustered regularly interspaced short palindromic repeats (CRISPR) associated nuclease 9) has revolutionized many areas of scientific research by aiding the development of advanced tools addressing certain limitations of traditional gene therapy approaches (Jie et al., 2015; Dai et al., 2016; Koo and Kim, 2016; Lino et al., 2018; Jacinto et al., 2020). Specifically, the development of inducible CRISPR-Cas9 transcriptional activator methods (CRISPRa) shows great potential toward studying the impact of upregulating genes that are involved in neuronal activity and synaptic function, particularly during disease states. These activator methods are based on the use of the nuclease-null (or “dead” dCas9) variants fused to transcriptional activator domains, allowing Cas9 to be used as a tool for modulate transcription activity (Gilbert et al., 2013; Maeder et al., 2013; Mali et al., 2013; Perez-Pinera et al., 2013; Chavez et al., 2015). The most popular of such CRISPR-Cas9 activator methods involves the use of VP64 (and combinations) transcriptional activator domains fused to the C-terminus of *Streptococcus pyogenes* (SP)-dCas9, and were shown to be able to increase endogenous expression (Gilbert et al., 2013; Mali et al., 2013; Perez-Pinera et al., 2013) of genes such as human *VEGFA* (Maeder et al., 2013), *L1RN*, *SOX2*, and *OCT4* genes (Cheng et al., 2013). However, the large size of such constructs only allowed their insertion into Lentiviral (LV) particles. These viral vectors have limited distribution from injection sites, when compared to the more widely-used Adeno Associated Virus (AAV). More recently, several other transcriptional activator domains have been described, such as VPR (Chavez et al., 2015), a tripartite effector composed of VP64, p65, and Rta transcription activator domains showing much increased induction of gene expression compared to traditional VP64-based approaches. Chavez et al. (2015) demonstrated the possibility to strongly activate the expression of *MIAT*, *NeuroD1*, *Ascl1*, *RhoxF2* genes using a dCas9-VPR activator system. In addition, an *Sp*dCas9-VPR based approach has been recently shown to be effective in inducing Brain Derived Neurotrophic Factor (BDNF) gene expression in neurons (Savell et al., 2019). But, as stated above, the limited coverage area associated with LV injections in the brain renders this approach less ideal for studies requiring delivery of target genes in larger brain areas.

To package a VPR-based activator system in AAV vectors, Ma et al. (2018) were able to modify the canonical *Sp*dCas9-VPR structure with a smaller ortholog from *Staphylococcus aureus* (*Sa*dCas9) and shorten the VPR domain. Although *Sp*dCas9 and *Sa*dCas9 derive from different bacterial species, when fused to VPR they induce the same transcriptional activator mechanisms. This alternative *Sa*dCas9-based system when delivered in several cell lines, was able to induce overexpression of several target genes.

In the recent years, various combinations of dCas9 proteins and transcriptional activators have been used successfully to increase gene expression *in vitro* and *in vivo* (Chavez et al., 2016; Zhou et al., 2018). Single and dual AAV vector systems have proven functional in overexpressing a variety of target genes, but the possibility of changing neurotransmission by altering the expression of synaptic proteins *in vivo* with CRISPRa has not been fully explored yet.

In this study, we added to the growing toolbox of CRISPRa systems by developing and verifying the performance of different *Sp*dCas9 and *Sa*dCas9-based activator approaches. To upregulate the expression of genes directly involved in neuronal and network activity, we aimed to adapt these technologies for the overexpression of *Cnr1*, the gene coding for Cannabinoid Receptor 1 (CB1), an endocannabinoid receptor expressed pre-synaptically in both excitatory and inhibitory neurons, responsible for feedback control of neurotransmitter release (Mackie, 2006).

## Materials and Methods

### Design of sgRNAs

The sgRNAs were designed to bind the promoter region of the mouse *Cnr1* gene. Using CHOPCHOP web tool^1^, we were able to select the optimum target site for CRISPR-Cas9 activation system. Four different sgRNAs were identified to bind 400 bp upstream to the transcription starting site (TSS) of the Cnr1 promoter. Each sgRNAs consist of 20-21 nucleotide followed by specific *Sa*Cas9 or *Sp*Cas9 sgRNA scaffolds. The sgRNAs sequences are provided in Supplementary Table 1.

### Screening of gRNAs

Approximately 16000 HEK293T cells/well were plated in 96 well-plates. After 6 h, cells were co-transfected with a mixture polyethyleneimine (PEI) transfection reagent and *Sa*dCas9-VPR, sgRNAs-Cnr1, CMV-BFP and Cnr1-tdTomato expressing plasmids with a DNA molar ratio of 2:1:1:0.1, respectively in a total of 200 ng per well using PEI:DNA ratio of 5:1. For the control conditions, cells were transfected using the same conditions described before and a random sgRNA expressing plasmid instead of sgRNAs-Cnr1 plasmid. Forty eight hours after co-transfection, cell well fixed with 1% PFA and the fluorescent intensity of BFP and tdTomato were measured. The fluorescent intensity of BFP, tdTomato, and the ratio tdTomato/BFP were normalized with the control condition.

### Molecular Cloning Methods

The molecular cloning methods (e.g., restriction digestion, ligation, and DNA electrophoresis in agarose gel) were performed according to standard procedures. DNA inserts and back bones were separately digested with restriction enzymes provided by Thermo Fisher Scientific and the ligation was performed according to the instruction provided by Anza T4 DNA ligase (Thermo Fisher Scientific). Zero Blunt TOPO PCR Cloning (Invitrogen) was used for the direct insertion of the sgRNAs-CNR1 blunt-end PCR product in a plasmid vector. The Golden Gate assembly method was used to accommodate the sgRNAs into the destination vector. The fragments were previously designed to have compatible 4 base overhangs in order to create circular vector (Engler et al., 2008; Potapov et al., 2018). The DNA was digested with restriction enzymes and the fragments were ligated with T4 DNA ligase (Thermo Fischer Scientific) in the same reaction. After the ligation reactions, the DNA was used to transform One Shot TOP10 (Invitrogen) or One Shot Stbl3 chemically competent bacteria (Invitrogen) using the heat-shock procedures. Plasmid DNA extraction and purification was done by using GeneJET Plasmid Miniprep Kit (Thermo Fischer Scientific) or PureLink^TM^ HiPure Plasmid Maxiprep Kit (Thermo Fisher Scientific) in other to obtain high number copies of transfection-grade plasmid. DNA fragments were isolated and purified from 1% agarose gel with the QIAquick Gel Extraction Kit (Qiagen).

### Lentivirus and AAV Productions

Recombinant lentiviruses were produced by co-transfection of HEK 293T cells with the transgene vector and the packaging plasmids pBR8.91 and pMD2G using the standard PEI method (Zufferey et al., 1997; Quintino et al., 2018). The lentivirus were harvested 48 h post-transfection and the pellet was obtained by ultracentrifugation of the medium containing lentivirus at 77,000 g for 90 min. Subsequently, the lentivirus were titered by infecting the HEK293T cells and after 72 h the DNA was extracted from the cells using DNeasy blood and tissue kit (Qiagen). qPCR was performed to amplify the woodchuck hepatitis virus post regulatory element (WPRE) and the human albumin (Alb). Relative quantification of WPRE and Alb expression was calculated by ΔΔCT method (Livak and Schmittgen, 2001). The resulting values were then used to estimate the titer of each lentiviral vector produced (Quintino et al., 2013, 2018; Supplementary Table 2).

Recombinant AAV vectors were produced by co-transfection of HEK 293T cells with the transgene plasmid and AAV8 plasmid from Plasmid Factory (pdp8), using PEI transfection method (Gray et al., 2011). The AAVs were harvested 72 h after transfection using polyethylene glycol 8000 (PEG8000) for the AAVs precipitation and chloroform for AAVs extraction (Davidsson et al., 2019). The AAV were collected and resuspended in PBS using Amicon Ultra-0.5 Centrifugal filters (Merck Millipore) (Wu et al., 2001). To calculate the number of genome-containing particles of the AAVs, we first made a standard curve using 10^2^–10^8^ copies of linearized plasmid. Subsequently, purified AAV vectors and standard curve DNA samples were quantified by qPCR using WPRE primers.

### Animals

P0-P1 animals of both sexes and 6–8 weeks old male C57BL/6 were used for *in vitro* and *in vivo* experiments, respectively. The experiments were conducted according to the Swedish Animal Welfare Agency as well as the international guidelines on the use of experimental animals.

### Primary Neuronal Isolation and Transduction

Cortical primary neurons were obtained from P0-P1 day old C57BL/6 mice. The entire brain was removed from the skull and the cortical areas were dissected in a petri dish containing cold Hibernate-E media supplemented with B-27 serum. The tissue was subsequently digested in a solution containing Papain (Sigma) and HBSS (Gibco) for 30 min at 37°C and a single cell resuspension was formed. The cells were centrifuged at 150 g for 5 min and the pellet was resuspended by adding 2 mL of Neurobasal medium supplemented with B27, Glutamax and Pen/Strep. 110,000 cells/well were plated on Poly-D-Lysine coated coverslips in 24-well plates and incubated at 37°C in 5% CO_2_. Half of the medium was changed 24 h after isolation and then every 2 days. Primary neurons were transduced with lentiviral viral vectors at a multiplicity of infection (MOI) 5 for a single viral vector transduction and MOI 10 for a dual viral vector transduction. 110,000 cortical primary neurons were also transduced with AAVs at an MOI of 10,000. Control cells transduced with vectors without sgRNAs (Empty) and non-treated cells were in some cases pooled and are referred to as “controls,” as no difference was found in any of the analyzed parameters.

### MEF Cell Culture and Transduction

Mouse Embryonic Fibroblast (MEF) were purchased from ATTC (ATCC^®^ SCRC-1040 TM). The cells were cultured according to the standard procedures provided from the company. For the cell’s transduction, approximately 50,000 cells were plated in a 6 well plate. After 3 h, the cells were transduced at a multiplicity of infection (MOI) 5 for a single viral vector transduction and MOI 10 (5 for each virus) for a dual viral vector transduction. As for primary neurons, control cells transduced with vectors without sgRNAs and non-treated cells were pooled and are referred to as “controls,” as no difference was found in any of the analyzed parameters.

### RNA Extraction and RT-qPCR

Animals used for RT-qPCR experiments were sacrificed by decapitation 3 weeks after lentivirus injection and 2 weeks after AAV injection. In correspondence to the injection site, the hippocampal hemisphere was removed and processed for total RNA extraction. Primary neurons an MEFs cultures were first washed with PBS to remove the residual medium, then there were processed for total RNA extraction. The RNA was extracted from hippocampal tissue, primary neurons and MEFs by Trizol reagent (Invitrogen) and purified using GeneJET RNA Purification Kit (Thermo Fisher Scientific), according to the manufacturer‘s instructions. On-column digestion of DNA was performed using the DNAse digestion Kit (Invitrogen). Approximately 70–300 ng of total RNA were retrotranscribed into cDNA using High-Capacity RNA-to-cDNA^TM^ Kit (Applied Biosystems) according to the manufacturer’s instructions provided. RT-qPCR using Taqman assay, was used to amplify the mouse *Cnr1*, *Cas9*, and *Gapdh* genes. The thermal cycle conditions included a denaturation cycle of 95°C for 10 min, followed by 40 cycle of amplification at 95°C for 15 s and 60°C for 1 min. The experiments of gene expression profiling were done in triplicate in three independent experiments. The results were analyzed using the 2-ΔΔCT method described by Livak and Schmittgen (2001) and the *Gapdh* gene was used to normalize the data.

### RNASeq

RNA libraries from primary neuronal cultures were generated starting from 15 ng of total RNA at 10 DIV, 7 days after virus transduction. RNA was processed using the SMART-Seq v4 Ultra low input RNA kit (Takara) followed by Nextera XT DNA library kit. Samples were analyzed on Nextseq500 using NSQ 500/550 Mid Output KT v2 (150 CYS).

### Western Blot

Total proteins were extracted using N-PER^TM^ Neuronal Protein Extraction agents (Thermo Fisher Scientific). After extraction, the lysate was subsequently centrifuged at 10,000 × g for 10 min at 4°C and the supernatant containing the proteins was used for protein quantification by Bradford assay. Five to twenty microgram of proteins were separated in a Bolt^TM^ 4–12% Bis-Tris precast polyacrylamide gel (Thermo Fisher Scientific) and subsequently transferred to PVDF membranes using a Trans-Blot Turbo Transfer system (BioRad). Membranes were incubated about 6 h with a TBST solution (10 mM Tris, pH 8.0, 150 mM NaCl, 0.5% Tween 20) supplemented with 5% skim milk (Sigma-Adrich). Membranes were washed three times for 10 min with a TBST solution and incubated over-night with the primary antibody anti-CB1 (Rabbit, Immunogenes, 1:1000 or Cayman Chemicals, 1:200) in TBST and 5% skim milk. Subsequently, the secondary antibody was added in a concentration of 1:1000 (anti-rabbit HRP; Abcam) for 2 h at room temperature. The blots were subsequently developed with the ECL substrate (Pierce^TM^ ECL western blotting substrate) according to the manufacturer’s protocols. After protein detection, the membrane was treated with a stripping solution (1M Tris-HCL, 20% SDS and β-Mercaptoethanol) and incubate with the anti-β-actin antibody conjugated with HRP (Sigma-Aldrich 1:50,000) for 1 h at room temperature. The blots were washed and developed with the ECL method described before. Image capture was performed with a ChemiDoc XRS + camera from BioRad. Expression levels were calculated with ImageLab software and normalized to β-Actin.

### Immunocytochemistry

Primary neurons were rinsed in PBS and rapidly fixed in 4% PFA for 15 min at room temperature (RT). Cells were permeabilized using a PBS solution containing 0.25% Triton X-100 (PBST) for 15 min at RT and then blocked for 2 h in PBST containing 5% Donkey serum. Primary antibodies were diluted in a PBST supplemented with 5% Donkey serum and incubated over night at 4°C. The primary antibody used were mouse anti-Map2 (Immunogene, 1:500) and rabbit anti HA-Tag (Thermo Fisher Scientific, 1:500). The secondary antibodies anti-mouse CY5 and anti-rabbit CY3 were added in a concentration of 1:500 an incubated for 2 h at RT in the dark. Primary neurons were washed with PBS for three times and subsequently loaded on a glass slide using a DABCO containing Hoechst diluted 1:1000. Images were taken on a Olympus BX51 upright fluorescent microscope. Quantification of markers was carried out manually by examining randomly three fields from three independent experiments and presented as percentage of double labeled cells.

### Lentiviral and AAV Injections in Mouse Hippocampus

C57BL/6 mice were anesthetized using ∼2.5% Isoflurane and subsequently fixed into a stereotactic frame. The lentiviral resuspension was injected into two different points according to the following coordinates: Medio-Lateral (ML) 2,9, Antero-Posterior (AP) -3,6, Dorso-Ventral (DV) -3,6 and -2,8 (from Dura) and ML 3,6; AP -3,2 and DV -3,5 and -1,5 (from Dura). The AAV resuspension was also injected into two different positions according to the following coordinates: AP -2,2, ML -1,7, DV-1,9 and -1,3 (from Dura) and AP -3,3, ML -3,0 and DV -3,7 and -2,7 (from Dura). Two different injection points were used to reach both dorsal and ventral hippocampus. 0,5 μL of lentiviral resuspension and 0,4 μL of AAVs resuspension were injected for each injection point at rate of 0,1 μL/min using a glass capillary.

### *In situ* Hybridization

The *in situ* hybridization was performed using the RNAScope method from Advanced Cell Diagnostics (ACD). According to the standard procedures, the mouse brain was removed from the skull and immediately frozen in liquid nitrogen. Before sectioning, the frozen brain was equilibrated for ∼1 h at −20°C. Subsequently, 15 μm thick sections were mounted onto Superfrost Plus Slides and kept drying at −20°C for 1 h. The slides were fixed in a chill 4% PFA for 15 min and immediately used for alcoholic dehydration procedure. The slides were kept drying at room temperature for 2 min before to proceed with the *in situ* hybridization protocol provided. The ZZ RNAScope probes used were the murine Cnr1, saCas9, EGFP, positive control Mm-Ppib and negative control DapB probes. The Supplementary Table 3 shows the details of the probes used in these experiments. TSA Plus Cyanine 5 (Cy5) or FITC detection kit in a concentration 1:750 were used for signal amplification. At the end of the procedures, approximately 4 drops of DAPI were added to each slide. The slides were quickly covered with a ProLong Antifade Mountant (Invitrogen) and closed using a glass coverslip. The samples were kept dry and processed for confocal imaging the day after using a Nikon A1 + confocal microscope equipped with 405, 488, 561, and 640 nm laser lines.

Analysis for quantification of RNAScope signals was performed using the Spot Detection algorithm in NIS Elements Advanced software (Nikon). Settings were selected to detect bright spots, clustered, with a typical diameter of 0.7 μm and contrast at 68.1. A region of interest (ROI) was drawn around the DG, CA3 and CA1 principal cell layers from images obtained with a 20 × 0.75 N.A. objective at Nyquist resolution (pixel size 0.14 μm), and detected spots were counted and normalized by the ROI area.

### Electrophysiology

Three to six weeks after viral vector injections, animals were briefly anesthetized with isofluorane and decapitated. The brains were quickly removed and immersed into ice-cold cutting solution, containing, in mM: sucrose 75, NaCl 67, NaHCO_3_ 26, glucose 25, KCl 2.5, NaH_2_PO_4_ 1.25, CaCl_2_ 0.5, and MgCl_2_ 7 (pH 7.4, osmolarity 305–310 mOsm). The cerebellum was discarded, the hemispheres were separated by a single midline cut and transverse 400 μm slices were cut with a vibratome (VT1200S, Leica microsystems). Three to four slices per hemisphere were collected and stored into a submerged recovery chamber filled with cutting solution for 30 min at 34°C, before being transferred to a second maintenance chamber filled with recording aCSF, containing in mM: NaCl 119, NaHCO_3_ 26, glucose 11, KCl 2.5, NaH_2_PO_4_ 1.25, CaCl_2_ 2, and MgSO_4_ 1.3 (pH 7.4, osmolarity 295–305 mOsm). All solutions were constantly bubbled with carbogen gas (95% O_2_ and 5% CO_2_).

For recordings, individual slices were transferred to a dual superfusion recording chamber (Supertech, Hungary) where they were perfused on both sides with recording aCSF at a speed of 2.5 mL/min/channel, heated to 38°C by an in-line heater placed 10–20 cm before the recording chamber and a bubble trap. This assures a constant temperature in the chamber of 34°C. GFP fluorescence was inspected by illuminating the slices with a 488 nm LED light source (Prizmatix, Israel) while observing the image through a camera. Only slices where GFP expression was deemed sufficient and covered the whole hippocampal formation were used for recordings. At this stage, some of the slices showing good GFP expression were immediately and quickly frozen in dry ice for western blot experiments.

Glass capillaries pulled from borosilicate glass and back-filled with recording aCSF were used as both stimulation and recording electrodes. Stimulating electrodes were placed in the middle portion of the stratum radiatum in CA1 to stimulate Schaffer collaterals originating from CA3 pyramidal neurons, and were connected to a current stimulator, where single 0.1 ms, 20–150 μA square pulses were delivered through the electrode every 15 s. Field excitatory post-synaptic potentials (fEPSPs) were monitored from the stratum radiatum of CA1 through the recording electrode connected to a EPC-10 amplifier (HEKA, Germany) and PatchMaster software (HEKA), and sampled at 20 kHz. Only recordings showing clear separation between the stimulation artifact, the pre-synaptic fiber volley and the fEPSP were used for further analysis. An input-output analysis was performed on each slice to assess slice quality, and slices responding with fEPSPs of <1 mV amplitude at maximal stimulation (typically 120–150 μA) were discarded. Baseline stimulation strength was set at 40% of the maximum. After recording a stable baseline period of 20 min, WIN 55,212-2 (Sigma-Aldrich, Sweden) was dissolved in DMSO and added to the perfusion aCSF to reach a final concentration of 1 μM and a DMSO dilution of at least 1:10,000. While recording baseline, DMSO without WIN in aCSF was used as recording solution.

After recordings, slices were fixed in 4% PFA overnight, and GFP expression was once again confirmed by fluorescence microscopy the day after.

### Statistical Analysis

All data are presented as the mean ± SEM, and *n* values indicate the number of independent experiments performed, the number of individual mice or slices. Significant differences were evaluated using an unpaired Student’s *t*-test with Welch’s correction or Mann-Whitney test where two groups were compared, or one-way analysis of variance (ANOVA) with Dunnett‘s *post hoc* test for multiple comparisons. Electrophysiological data was analyzed with two-way repeated measures ANOVA followed by Sidak’s multiple comparison test to test for differences between time points over time. Statistical analyses were performed using Prism software (GraphPad). Group differences were considered statistically significant at ^∗^*p* < 0.05.

## Results

### *Sp*dCas9 Based Lentiviral System for the Spatial Control of *Cnr1* Gene Expression

**FIGURE 1:**
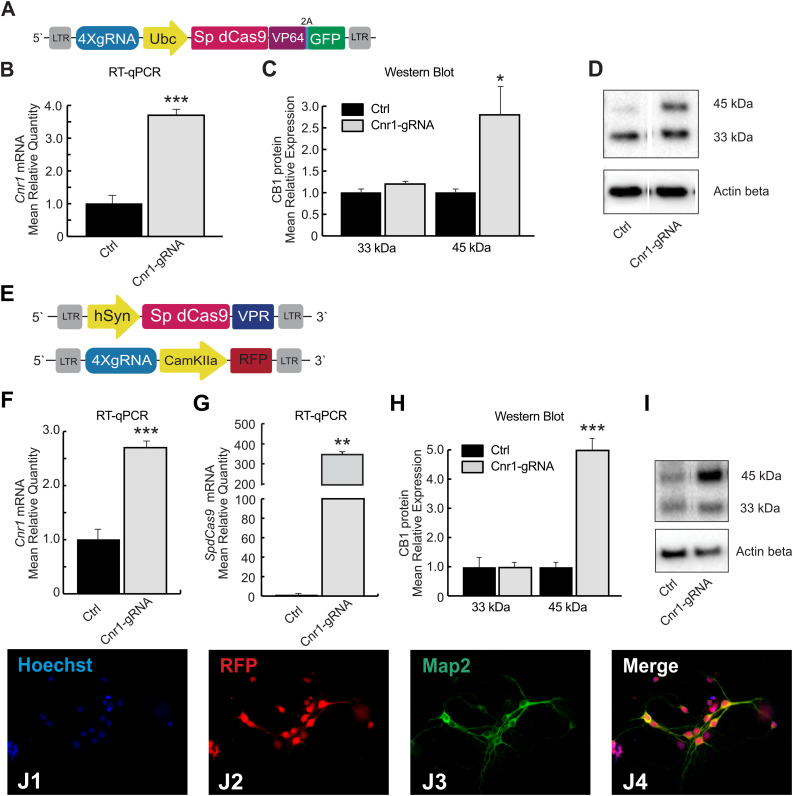
*Sp*dCas9 based lentiviral system and modulation of *Cnr1* gene expression *in vitro*. **(A)** Schematic illustrating the main components of the single lentiviral vector LV-*Sp*dCas9-VP64-Cnr1. **(B)** mRNA expression level of the *Cnr1* gene in primary neurons transduced with the single lentiviral vector LV-*Sp*dCas9-VP64-Cnr1 (Cnr1-gRNA) compared with controls (Ctrl) (Unpaired Student’s *t*-test, ***p < 0.001). **(C)** Expression level of the two immunoreactive CB_1_ bands (∼33 and 45 kDa) in primary neurons (Cnr1-gRNA vs. Ctrl, Unpaired Student’s *t*-test, *p < 0.05). **(D)** WB analyses of total protein lysates from primary neurons. The upper panel shows the detection of the immunoreactive CB_1_ bands (∼33 and 45 kDa) and the lower panel shows β-actin detection for loading control. **(E)** Schematic of the main components of the dual viral vector system LV-*Sp*dCas9-VPR and LV-Cnr1-gRNA. **(F)** mRNA expression level of the *Cnr1* gene in primary neurons transduced with the dual viral vector system LV-*Sp*dCas9-VPR and LV-Cnr1-gRNA (Cnr1-gRNA) compared with controls (Ctrl) (Unpaired Student’s *t*-test, ***p < 0.001). **(G)** mRNA expression level of the *spdCas9* gene in primary neurons (Unpaired Student’s *t*-test, **p < 0.01). **(H)** Expression level of the two immunoreactive CB_1_ bands (∼33 and 45 kDa) in primary neurons (Cnr1-gRNA vs. Ctrl, Unpaired Student’s *t*-test, ***p < 0.001). **(I)** WB analyses of total protein lysates from primary neurons. **(J1–J4)** Representative micrographs obtained with fluorescence microscopy of cortical primary neurons. The images show the Hoechst nuclear staining (Blue; **J1**), RFP expression (Red; **J2**) and GFP (Green; **J3**) expression. Scale bar: 80 μm.

We first generated two different Lentivirus-based CRISPR-dCas9 activator systems able to induce spatial control of *Cnr1* expression in mammalian target cells (Figures 1A,E). Both technologies are based on guide RNAs components, targeting the promoter region of the *Cnr1* gene, and the inactivated version of the Cas9 enzyme derived from *Streptococcus pyogenes* (*Sp*dCas9). The first system consists in a single vector carrying *Sp*dCas9 fused to the transcriptional activator domain VP64, a reporter gene GFP separated by a 2A sequence and four sgRNAs (LV-*Sp*dCas9-VP64-Cnr1, Figure 1A). The general promoter UbC drives the expression of *Sp*dCas9-VP64-2A-GFP, while the four gRNAs are driven by the human or murine hH1, h7SK, hU6 and mU6 promoters (Figure 1A). The second system is composed by *Sp*dCas9 fused to the transcriptional activator domain VPR in one vector (driven by the human Synapsin promoter, LV-*Sp*dCas9-VPR), and sgRNA components together with a reporter gene GFP in a second vector (LV-Cnr1-gRNA, Figure 1E).

We first evaluated constructs efficiency in MEFs cultures that do not normally express high level of *Cnr1*. MEFs were transduced with lentivirus carrying the single construct and after 72 h we validated gene activation by RT-PCR. Gene expression analysis showed a significant upregulation of *Cnr1* gene in MEFs transduced with LV-*Sp*dCas9-VP64-Cnr1 compared with controls (Fold Change (FC) 50.7 ± 0.57; Ctrl vs. Cnr1-gRNA; *p* < 0.001; *n* = 6) (Supplementary Figure 1A).

To evaluate the efficiency of the single lentiviral vector in neurons, we used cortical primary neurons from early post-natal mice. Primary neurons were transduced with lentivirus 3 days after neuronal isolation (DIV 3) and 10 days after, we validated the efficiency of the vector by RT-PCR and WB analysis. Gene expression profiling using RT-PCR revealed a significant upregulation of *Cnr1* gene in cortical primary neurons transduced with the LV-*Sp*dCas9-VP64-Cnr1, compared with controls (FC 3.6 ± 0.17; Ctrl vs. Cnr1-gRNA; *p* < 0.001; *n* = 6) (Figure 1B). Western blot analysis revealed two immunoreactive CB_1_ bands in primary neurons. The most prominent immunoreactive band had a molecular mass of ∼33 kDa and the additional band a molecular mass of ∼45 kDa. Protein expression analysis showed an increased expression of the immunoreactive CB_1_ band with a molecular mass ∼45 kDa in primary neurons transduced with LV-*Sp*dCas9-VP64-Cnr1, compared with controls (FC 2.7 ± 0.82; Ctrl vs. Cnr1-gRNA ∼45 kDa, *p* < 0.05, *n* = 6 and FC 1.2 ± 0.36, Ctrl vs. Cnr1-gRNA ∼33 kDa; *p* > 0,05; *n* = 6) (Figures 1C,D).

Taken together, these data show that using this strategy, we were able to increase the expression of CB1 receptors in MEF and in primary neurons at both mRNA and protein levels.

To ensure the highest possible expression levels of both *Sp*dCas9 and sgRNAs components, and selectively direct *Sp*dCas9 expression into neurons, we designed instead a dual lentiviral vector system (Figure 1E). Compared to the single-vector strategy, this approach involves the transcriptional activator domain VPR that it is noted to greatly increase gene expression compared to VP64 domain (Chavez et al., 2015). To validate the performance of the dual-vector system in neurons, primary neuronal cells from postnatal mice were transduced with LV-*Sp*dCas9-VPR and LV-Cnr1-gRNA at DIV3, and RT-PCR, WB and immunofluorescent (IF) analysis was performed 10 days after.

Similar to the single-vector system, RT-PCR analysis showed a significant upregulation of the *Cnr1* gene in cortical primary neurons transduced with the vector combination compared with controls (FC 2.7 ± 0.07; Ctrl vs. Cnr1-gRNA; *p* < 0.001; *n* = 6) (Figure 1F). Using the same method, we also evaluated the *Sp*dCas9 gene expression and detected a significant presence of *Sp*dCas9 in primary neurons transduced with LV-*Sp*dCas9-VPR and LV-Cnr1-gRNA compared with non-treated controls (FC 342.0 ± 50.9; Ctrl vs. Cnr1-gRNA; *p* < 0.001; *n* = 6) (Figure 1G).

In parallel, WB analysis showed an increased expression of the immunoreactive CB_1_ band with a molecular mass ∼45 kDa in treated primary neurons compared with controls (FC 4.98 ± 0.12; Ctrl vs. Cnr1-gRNA ∼45 kDa; *p* < 0.001; *n* = 6 and FC 0.96 ± 0.25 Ctrl vs. Cnr1-gRNA ∼33 kDa; *p* > 0,05; *n* = 6) (Figures 1H,I), and IF analysis revealed co-localization of the mature neuronal marker Map2 and RFP (Figures 1J1–4), indicating that the constructs were expressed correctly in mature neurons.

These results indicate that the dual lentiviral viral vector system can infect primary neurons and most importantly is able to upregulate *Cnr1* gene expression at mRNA and protein level. Using these strategies, we were able to continuously express *Sp*dCas9 and modulate *Cnr1* gene expression immediately after the viral vector treatment.

### *Sp*dCas9 Based Lentiviral System for the Temporal Control of *Cnr1* Gene Expression

Specific gene expression modulation in neuronal cell populations could be a powerful tool to analyze the mechanisms underlying neuronal circuits, and the CRISPR-Cas9 based technologies described above are able to continuously upregulate the expression of *Cnr1* in all neurons. However, *Cnr1* expression in the brain is highly dynamic and plastic, and differently responds to distinct network states. *Cnr1* expression is for example highly and consistently downregulated during the early stages of epileptogenesis, and therefore designing an overexpression system with precise temporal control might be more appropriate when considering CRISPR/Cas9 gene therapy for disease modification.

**FIGURE 2:**
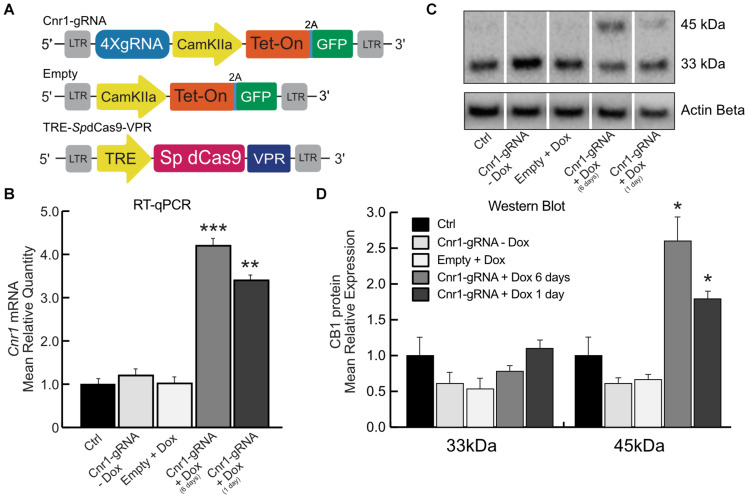
Doxycycline-inducible *Sp*dCas9-VPR system. **(A)** Schematic illustrating the main components of the DoxCRISPR-Cas9 dual lentiviral system LV-TRE-*Sp*dCas9-VPR, LV-Cnr1-gRNA-rtTA-GFP, and LV-Empty. **(B)** The bar graphs show the mRNA expression level of the *Cnr1* gene in primary neurons transduced with the DoxCRISPR-Cas9 dual lentiviral system at two different time points (Cnr1-gRNA + Dox 1 and 6 days), compared to non-transduced (Ctrl), non-treated (Cnr1-gRNA -Dox) controls and cells transduced with LV-Empty and receiving Dox (Empty + Dox) (One-way ANOVA followed by Dunnett’s multiple comparison test, **p < 0.01 and ***p < 0.001). **(C)** WB analyses of total protein lysates from primary neurons. The upper panel shows the detection of the immunoreactive CB_1_ bands (∼33and 45 kDa) and the lower panel shows β-actin detection for loading control. **(D)** Expression level of the two immunoreactive CB_1_ bands (∼33 and 45 kDa) in primary neurons (One-way ANOVA followed by Dunnett’s multiple comparison test, *p < 0.05).

Toward this goal, we generated a dual lentiviral vector CRISPR-Cas9 system for temporal control of *Sp*dCas9-VPR and *Cnr1* gene expression. In these constructs, the *Sp*dCas9-VPR transgene is under the control of the Tetracycline Responsive Element (LV-TRE-*Sp*dCas9-VPR), while in the second vector the promoter CamKIIa1.3 controls expression of the Doxycycline-sensitive transcriptional activator rtTA and the reporter gene GFP, separated by a 2A sequence (LV-Cnr1-gRNA-rtTA-GFP). This second vector also expresses four sgRNAs under the control the human or murine hH1, h7SK, hU6 and mU6 promoters (Figure 2A). With this strategy, *Sp*dCas9-VPR is expressed only during periods of Doxycycline (Dox) administration.

We evaluated the efficiency of the DoxCRISPR-Cas9 dual lentiviral system in both MEFs and in cortical primary neurons under different conditions of Dox administration. Dox was administered for 1 or 6 days after lentiviral co-transduction, and *Cnr1* gene upregulation was evaluated by RT-PCR and WB analysis. RT-PCR showed a significant upregulation of *Cnr1* in MEFs after 1 day (FC 7.38 ± 0.73; Cnr1-gRNA + Dox 1 day; *p* < 0.05; *n* = 6) and 6 days (FC 10.04 ± 0.72; Cnr1-gRNA + Dox 6 days; *p* < 0.001; *n* = 6) exposure to Dox compared to both non-transduced (Ctrl) and non-treated (-Dox) controls, or cells transduced with an empty gRNA vector and exposed to Dox (Empty + Dox, Supplementary Figure 1B). Similarly, RT-qPCR showed a significant upregulation of *Cnr1* gene (FC 4.22 ± 0.43; Ctrl vs. Cnr1-gRNA + Dox 6 days; *p* < 0.001, *n* = 6; FC 3.25 ± 0.33; Ctrl vs. Cnr1-gRNA + Dox 1 day; *p* < 0.001, *n* = 6) (Figure 2B) in cortical primary neurons.

CB1 protein expression analysis by WB in primary neurons showed the same expression patterns obtained in the single and dual lentiviral vector system described before. The analysis showed an increased expression of the immunoreactive CB_1_ band with a molecular mass ∼45 kDa in samples co-transduced with LV-TRE-*Sp*dCas9-VPR and LV-Cnr1-gRNA-rtTA-GFP by 1 and 6 days exposure to Dox (FC 2.58 ± 0.18 Ctrl vs. Cnr1-gRNA + Dox 6 days ∼45 kDa; *p* < 0.05, *n* = 3; FC 1.72 ± 0.21 Ctrl vs. Cnr1-gRNA + Dox 1 day ∼45 kDa; *p* < 0.05, *n* = 3) (Figures 2C,D).

Taken together, these data show that it is possible to achieve temporal control of *Cnr1* gene expression by Dox administration, with significant increases in both mRNA and protein levels with as little as 1 day of Dox exposure.

### *Sp*dCas9-VPR Expression in the Mouse Hippocampus

Our *in vitro* data shows that we are able to induce expression of CB1 receptors in neuronal cultures, but to potentially apply this technology for disease modeling or modulation, we needed to confirm its function also *in vivo*. We therefore injected adult C57BL/6J mice with the combination of LV-*Sp*dCas9-VPR and LV-Cnr1-gRNA, previously described in Figure 1E, in the hippocampus. After 3 weeks, we analyzed the mouse brains for both histological and molecular evaluations.

**FIGURE 3:**
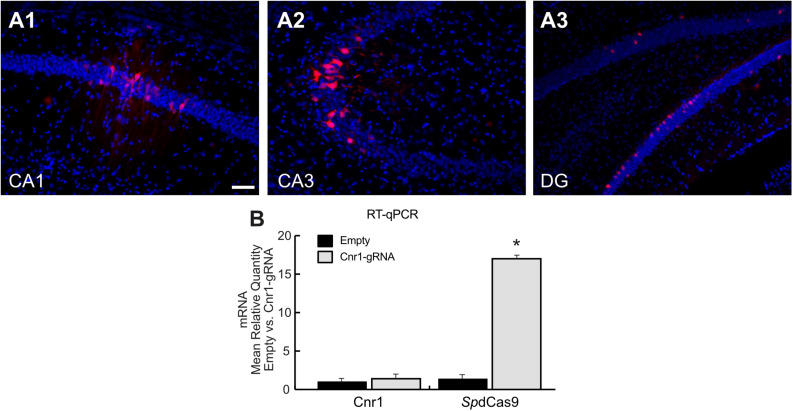
Histological and molecular evaluation of *sp*dCas9-VPR and *Cnr1* expression in the mouse hippocampus. **(A1–A3)** The images show coronal sections (30 μm) of mouse hippocampus injected with the combination of LV-*Sp*dCas9-VPR and LV-Cnr1-gRNA. Representative images of RFP expression (Red) and Hoechst nuclear staining (Blue) in CA1 **(A1)**, CA3 **(A2)**, and DG **(A3)**. Scale bar: 50 μm. **(B)** The bar graphs show the mRNA expression level of the *Cnr1* and *spdCas9* genes in mouse hippocampus (Cnr1-gRNA vs. Empty, Mann-Whitney test, *p < 0.05).

Histological analysis of hippocampal slices showed only a very limited expression of RFP in all major principal cell layers (Figure 3A), and RT-PCR showed no significant upregulation of the *Cnr1* gene in the injected hippocampi compared with non-treated controls (FC 1.43 ± 0.43; *p* > 0.05; *n* = 4), despite *Sp*dCas9 being expressed at significant levels (*p* < 0.05; *n* = 4) (Figure 3B).

These data suggest that although the dual viral vector system is able to increase the expression of the neuronal gene in *in vitro* models such as in primary neurons and MEF, the same system is not able to upregulate *Cnr1* gene expression in *in vivo* mouse brain.

### Generation of S*a*dCas9-VPR Viral Vector Technology for the Overexpression of *Cnr1* Gene

The limited hippocampal expression of the lentivirus based *Sp*dCas9 system *in vivo* prompted us to refine this technology for a better translation into animal models. Lentiviruses have the advantage of a large transgene capacity, but their spread into brain tissue outside of the injection site is limited. In addition, after transduction, the transgene is integrated randomly into the host cell genome, with unpredictable outcome on potential integration on silenced genomic regions. To ensure the highest possible expression levels of dCas9-VPR and sgRNAs, as well as optimize the spread of viral vector in target brain areas, we assembled different constructs for Adeno-Associated vectors (AAVs). The main limitation of AAV vectors consists on their limited packaging capacity, which is around 4.8 kb, including 300 bp of their Inverted Terminal Repeats (ITRs). Since the size of the *Sp*dCas9 transgene is substantial (4.1 kb) there is limited space left for promoter and the VPR transcriptional activator on the same vector. The use of a smaller dCas9 variant is therefore necessary. A recent report has described the development of a “mini-dCas9-VPR” variant based on the dCas9 protein from *Staphylococcus aureus* (Sa) and a modified, shortened version of the VPR activator (Ma et al., 2018). We adapted this system and integrated it with a second vector carrying the necessary gRNAs and a reporter gene. Our modified CRISPRa *Sa*dCas9-VPR vector system is composed of two vectors: first, the *Sa*dCas9 variant fused to the transcriptional activator domain VPR under the control of the human Synapsin 1 gene promoter (hSyn1), to confer highly neuron-specific transgene expression (Kügler et al., 2003) as well as the hemagglutinin tag (HA-tag) for construct expression validation trough immunohistochemical analysis (AAV-*Sa*dCas9-VPR). Preliminary data (data not shown) suggested that the nuclear import of the mini VPR system was suboptimal in neurons. Therefore, we further reengineered the mini-dCas-VPR system by removing the original nuclear localization signals and adding synthetic ones (Ma et al., 2018). Second, the sgRNA vector contained a reporter gene GFP under the control of the CamKIIa1.3 promoter to selectively identify the expression in the excitatory neurons and up to 4 sgRNA designed selectively to target the promoter region of the *Cnr1* gene (AAV-Cnr1-gRNA). Each sgRNA is expressed separately as earlier, and driven by hH1, h7SK, hU6 and mU6 promoters. Lastly, we also designed a non-targeting control vector using the same AAV-CamKIIa-GFP construct but without the sgRNAs (AAV-Empty) for the determination of baseline cellular responses in the different experimental conditions (Figure 5A).

### Validating sgRNAs for the *Sa*dCas9 CRISPRa System

**FIGURE 4:**
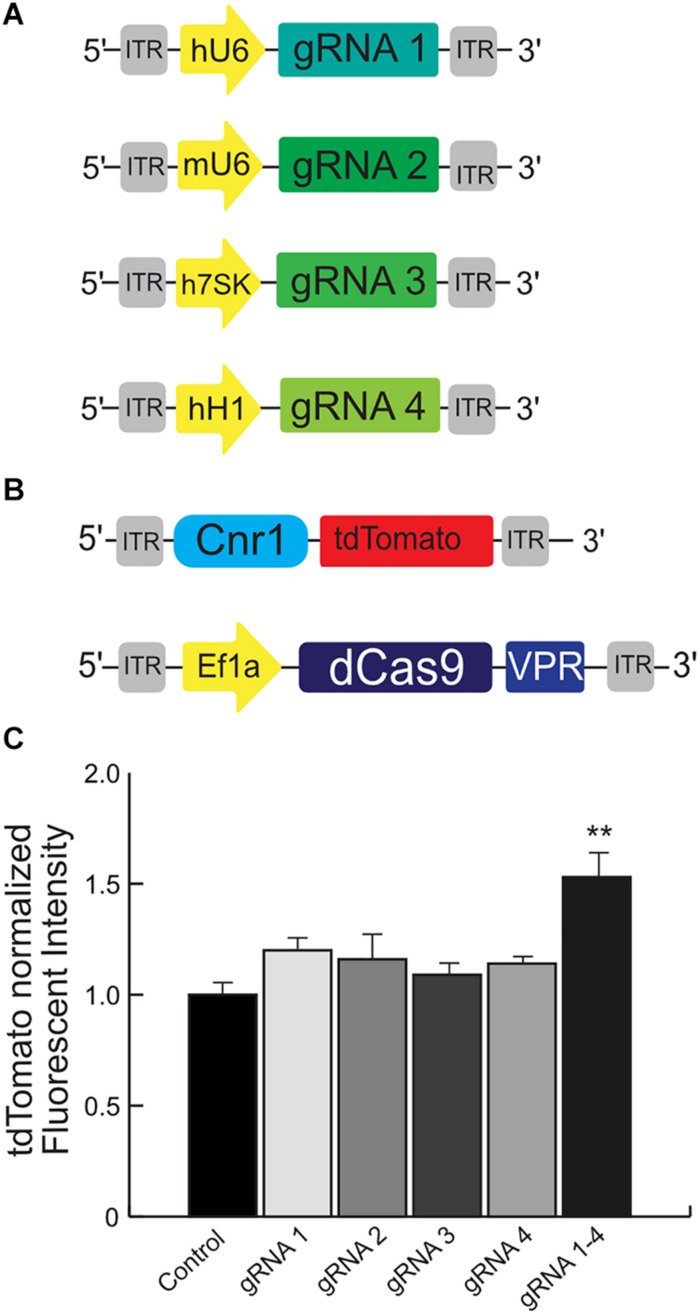
Schematic illustrating the main components used for the validation of the mouse Cnr1-sgRNA for the CRISPR-Cas9 transcriptional activator system. The illustration **(A)** show the plasmids carrying four independent sgRNAs to selectively bind the mouse promoter *Cnr1*. The illustration **(B)** show the plasmid carrying the target mouse genomic sequence Cnr1 and the plasmid coding the *Sa*dCas9-VPR. **(C)** The combination of all four independent sgRNAs can induce a significant up-regulation of tdTomato intensity. (One-way ANOVA followed Dunnett’s multiple comparison test, gRNA vs. Ctrl, **p < 0.01) compared to single sgRNAs.

We engineered four independent sgRNA to selectively bind the mouse promoter *Cnr1*, using the *in silico* tool CHOPCHOP^2^ and selected the best sgRNAs based on their predicted scores for specificity to minimize off-target binding (Figure 4A). However, although *in silico* methods such as these are highly efficient, the validation of individual sgRNAs is still necessary, especially for a system based on *Sa*dCas9.

We used HEK293T cells transfected with several plasmids: one is carrying the target mouse genomic sequence *Cnr1* (−500 to −50 bp from the TSS), ahead of tdTomato; the second is coding *Sa*dCas9-VPR; the third carries CMV-BFP for normalization of expression levels and the last plasmids carry individual sgRNAs (Figures 4A,B, sequences in Supplementary Table 1). An increase in tdTomato intensity is used as a performance marker for single sgRNAs, meaning that the particular sgRNA is capable of binding to the *Cnr1* genomic sequence ahead of tdTomato and drive expression of the fluorescent protein.

Using this tool, we detected a significant up-regulation of tdTomato intensity when cells were co-transfected with the combination of all four sgRNAs (sgRNA 1/2/3 and 4 vs. all individual sgRNA1–4; FC 1.53 ± 0.08, *p* < 0.05; *n* = 4), but not with individual sgRNAs (Figure 4C). These results suggest that the engineered sgRNA are able to bind the target mouse genomic sequence before the *Cnr1* TSS and modulate the expression of the reporter gene tdTomato, but only when acting in concert. This is in line with other reports showing a synergistic action of multiple sgRNAs acting on the same genomic target (Cheng et al., 2013).

### *Sa*dCas9-VPR Induces an Overexpression of *Cnr1* Gene in Cortical Primary Neurons

**FIGURE 5:**
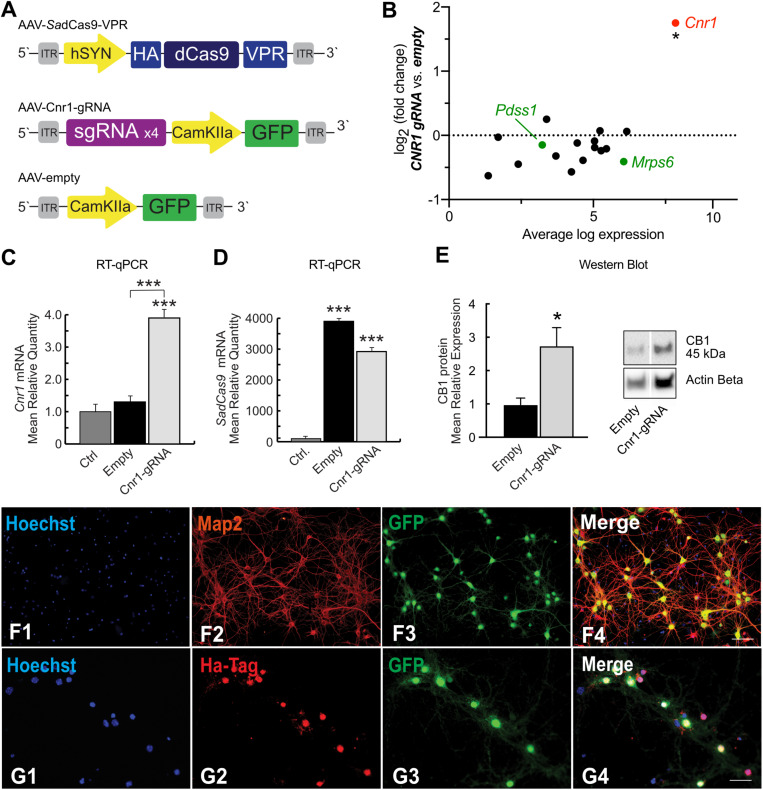
*Sa*dCas9-VPR based AAV vector system and modulation of *Cnr1* gene expression in cortical primary neurons. **(A)** Schematic illustrating the main components of the CRISPRa *Sa*dCas9-VPR vectors system. The first vector expresses the *Sa*dCas9 variant fused to the transcriptional activator domain VPR under the control of the hSyn1 promoter (AAV-*Sa*dCas9-VPR), the second vector carrying the individual sgRNAs contains the reporter gene GFP under the control of the CamKIIa1.3 promoter (AAV-Cnr1-gRNA), and the third vector represents the empty control (AAV-empty). **(B)** Fold change expression in mRNA levels plotted against baseline expression for potential off-target genes where sgRNAs designed for *Cnr1* bind within 2kb of the TSS (black) or within promoter regions (green) (Unpaired *t*-test *p < 0.05). **(C)** mRNA expression levels of the *Cnr1* gene in primary neurons transduced with AAV-*Sa*dCas9-VPR and AAV-Cnr1-gRNA (Cnr1-gRNA) compared with non-treated controls and empty controls (Empty) (One-way ANOVA followed Dunnett’s multiple comparison test, ***p < 0.001). **(D)** mRNA expression level of the *saCas9* gene in primary neurons (One-way ANOVA followed Dunnett’s multiple comparison test, ***p < 0.001). **(E)** Total protein western blot from primary neurons showing increased CB1 expression in cells treated with AAV-*Sa*dCas9-VPR and AAV-*Sa*dCas9-VPR (Cnr1-gRNA) compared to Empty vector controls (Cnr1-gRNA vs. Empty, Mann-Whitney test, *p < 0.05). **(F1–F4,G1–G4)** Representative micrographs obtained with fluorescence microscopy of cortical primary neurons transduced with CRISPRa *Sa*dCas9-VPR vectors system to up regulate *Cnr1* gene expression. **(F1–4)** The images show the Hoechst nuclear staining (Blue; **F1**), Microtubule associate protein 2 staining (Map2; Red; **F2**) and GFP (Green; **F3**) expression. Scale bar: 50 μm. **(G1–G4)** The images show the Hoechst nuclear staining (Blu; **G1**), Hemagglutinin Tag staining (Ha-Tag; Red; **G2**) and GFP (Green; **G3**) expression. Scale bar: 80 μm.

To evaluate the specificity and the efficiency of the dual AAV viral vector system (Figure 5A) on *Cnr1* gene expression, we first validated both constructs in an *in vitro* model, as earlier. Cortical primary neurons were transduced at DIV3 with a combination of AAV-*Sa*dCas9-VPR and AAV-Cnr1-gRNA or AAV-empty. Seven days after we evaluated vectors specificity and efficiency by next generation sequencing (NGS), RT-qPCR and ICC.

To evaluate the specificity of our designed sgRNAs (packaged in AAV-Cnr1-gRNA) in targeting only the promoter region of the *Cnr1* gene in the mouse genome, we first determined potential off-target sites with a maximum of four base pair mismatch using the online CasOFF Finder tool (Bae et al., 2014). This returned a list of 171 potential off-target sites (full list in Supplementary Table 4), of which 14 were within 2 kb of a TSS and only 2 were in a promoter region, specifically in the promoter of *Pdss1* and *Mrps6* (Figure 5B, green dots). RNASeq analysis from primary neurons showed that none of these 16 genes were significantly altered in cells treated with the AAV-*Sa*dCas9-VPR and AAV-Cnr1-gRNA vector combination, but indicated that *Cnr1* was significantly upregulated (Figure 5B).

To further confirm *Cnr1* upregulation, we performned RT-qPCR on a separate set of cultures, and confirmed a significant upregulation of *Cnr1* in cortical primary neurons transduced with AAV-*Sa*dCas9-VPR and AAV-Cnr1-gRNA compared with non-treated controls (Ctrl vs. AAV-gRNA-Cnr1; FC 3.92 ± 0.45, *p* < 0.0001; *n* = 9) and with the non-targeting controls (AAV-Empty; *p* < 0.0001; *n* = 9, Figure 5C). RT-qPCR also confirmed the presence of *Sa*dCas9 in the cortical primary neurons transduced with AAV-*Sa*dCas9-VPR compared with non-treated controls (Ctrl vs. AAV-gRNA-Cnr1 and Ctrl vs. Empty, *p* < 0.0001, *n* = 6, Figure 5D). Western blot experiments further showed that also CB1 protein levels were increased in cells treated with the AAV-gRNA-Cnr1 vector compared to AAV-Empty (FC 2.73 ± 0.55, *p* < 0.05, *n* = 5, Figure 5E).

Immunocytochemistry analysis revealed co-localization of mature neuronal marker Map2 and GFP in cortical primary neurons transduced with AAV-*Sa*dCas9-VPR and AAV-Cnr1-gRNA, where 96.31% of primary neurons expressing Map2 co-localized with GFP. These results indicate that the vectors are able to infect mature excitatory neurons (Figures 5F1–4). ICC analysis also revealed a high co-localization of HA-Tag and GFP (86.59% of GFP positive neurons co-localized with the HA-Tag), indicating that *Sa*dCas9 protein is expressed in targeted neurons. These results show additionally that AAV-*Sa*dCas9-VPR and AAV-Cnr1-gRNA (GFP) are expressed in the same neuronal population confirming the efficiency and applicability of this dual viral vector system (Figures 5G1–4).

### *Sa*dCas9-VPR and *Cnr1* Expression in the Mouse Hippocampus

**FIGURE 6:**
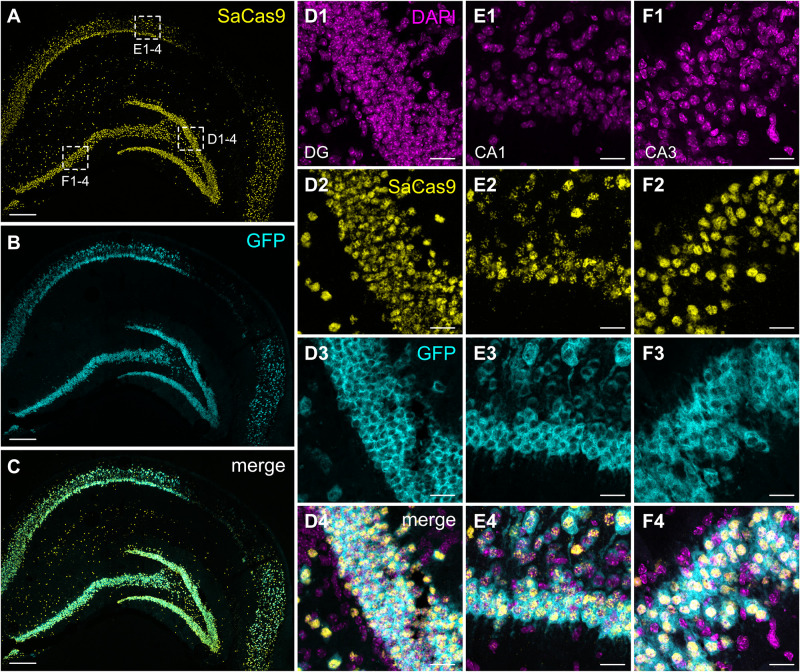
Expression of *Sa*dCas9 and GFP mRNAs in the mouse hippocampus. *In situ* hybridization was performed with a two RNAscope probes targeting *Sa*dCas9 and GFP mRNA. The images show coronal sections (15 μm) of mouse hippocampus injected with AAV-*Sa*dCas9-VPR and AAV-Cnr1-gRNA. Representative images for *Sa*dCas9 (**A**; Yellow dots), GFP mRNA (**B**; Cyan dots) and DAPI (**C**; Magenta dots). High magnification images show *Sa*dCas9 and GFP mRNA expression in the DG **(D1–D4)**, CA1 **(E1–E4)** and CA3 **(F1–F4)**. Scale bar in **(A–C)** is 250 μm, **(D–F)** 25 μm.

After confirming the functionality of our system in primary neurons, we injected the same mixture of AAVs into the hippocampus of adult mice. Histological evaluation of hippocampal slices revealed extensive expression of GFP throughout all hippocampal layers, as well as significant dorso-ventral coverage. Because of the lack of well-performing antibodies against *Sa*Cas9 or the HA-tag in slices, we used a dual RNAscope *in situ* hybridization to simultaneously evaluate the expression of the *Sa*dCas9 and GFP mRNAs *in vivo*. Probing with a specific probe against *Sa*dCas9 revealed expression of the mRNA in all principal cell layers of the hippocampus, as well as in putative interneurons (Figure 6) only on the ipsilateral side, confirming that our AAV-*Sa*dCas9-VPR can efficiently infect target neurons and induce expression of the *Sa*dCas9 mRNA. In addition, GFP mRNA expression was also seen in all principal layers of the hippocampus (GFP in the second vector is driven by the CaMKIIa promoter), and the GFP and *Sa*dCas9 *in situ* signals were overlapping in virtually all expressing cells (*Sa*dCas9 expression in GFP positive cells: 97.5 ± 2.1% in DG, 98.2 ± 1.8% in CA1 and 97.9 ± 2.1% in CA3, Figure 6), indicating the simultaneous expression of both AAVs in all transduced cells also *in vivo*.

CB1 receptors are expressed on both excitatory and inhibitory synaptic terminals, but at very different levels. While their expression and localization on pre-synaptic boutons in GABAergic terminals is easily visualized by immunohistochemistry, their expression level on glutamatergic synapses is very low and cannot be reliably visualized and measured by antibody-based techniques (Marsicano and Lutz, 1999; Katona, unpublished). We therefore applied the RNAscope technology also for visualizing and quantifying the amount of *Cnr1* mRNA *in situ* in hippocampal slices from animals injected with our vector combinations.

**FIGURE 7:**
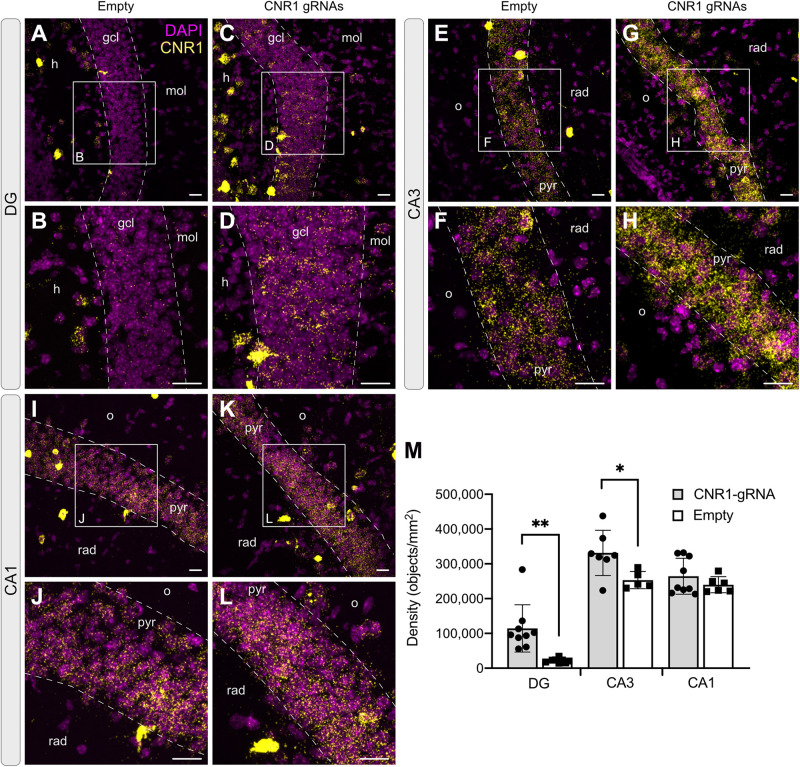
Expression of the Cnr1 mRNA in the mouse hippocampus. *In situ* hybridization was performed with the RNAscope probe targeting Cnr1 mRNA. The images show coronal sections (15 μm) of mouse hippocampus injected with AAV-*Sa*dCas9-VPR and AAV-Cnr1-gRNA (Cnr1-gRNA) or AAV-*Sa*dCas9-VPR and AAV-empty (Empty). Representative images for Cnr1 mRNA (Yellow dots) and Dapi (Magenta dots) in the DG **(A–D)**, CA3 **(E–H)**, and CA1 **(I–L)**. Scale bars are 25 μm. gcl: granular cell layer; mol: molecular layer; h: hilus; o: stratum oriens; pyr: pyramidal layer; rad: stratum radiatum. **(M)** Bar graphs showing the quantification of Cnr1 mRNA signal expressed in density of particles per mm^2^ in the DG, CA3, and CA1 hippocampal regions of the experimental groups CNR1 gRNAs and Empty. *N* = 5–9 slices from 3 animals per group. (Cnr1-gRNA vs. Empty, Unpaired *t*-test ^∗∗^*p* < 0.01; *p < 0.05).

In animals injected with AAV-*Sa*dCas9-VPR and AAV-empty, *Cnr1* mRNA was easily visualized in all principal layers of the hippocampus, with higher expression in CA1 and CA3 compared to the DG, where only few positive signals were detected in granule cells (Figure 7). This is according to previously published reports showing that DG mossy fibers are less sensitive than Schaffer collaterals to CB1 receptor agonists (Hofmann et al., 2008; Caiati et al., 2012). In animals injected with the AAV-Cnr1-gRNA vector and AAV-*Sa*dCas9-VPR, a significant fivefold upregulation of *Cnr1* mRNA was observed particularly in DG granule cells (from 21,549 ± 2,257 in AAV-Empty to 114,343 ± 22,675 in AAV-Cnr1-gRNA, density of particles per mm^2^, *p* < 0.05, Figures 7A–D,M), but also to a lesser extent in the CA3 area (from 253,322 ± 11,107 to 331,531 ± 24,530, *p* < 0.05, Figures 7E–H,M). Quantification of *Cnr1* mRNA signal on putative interneurons was not possible due to the very high basal level of expression even in control conditions.

Taken together, these data show that our dual AAV-based system can be successfully expressed in the brain of adult animals and is able to significantly upregulate the expression of *Cnr1*.

### Functional Evaluation of *Cnr1* Upregulation in the Hippocampus

**FIGURE 8:**
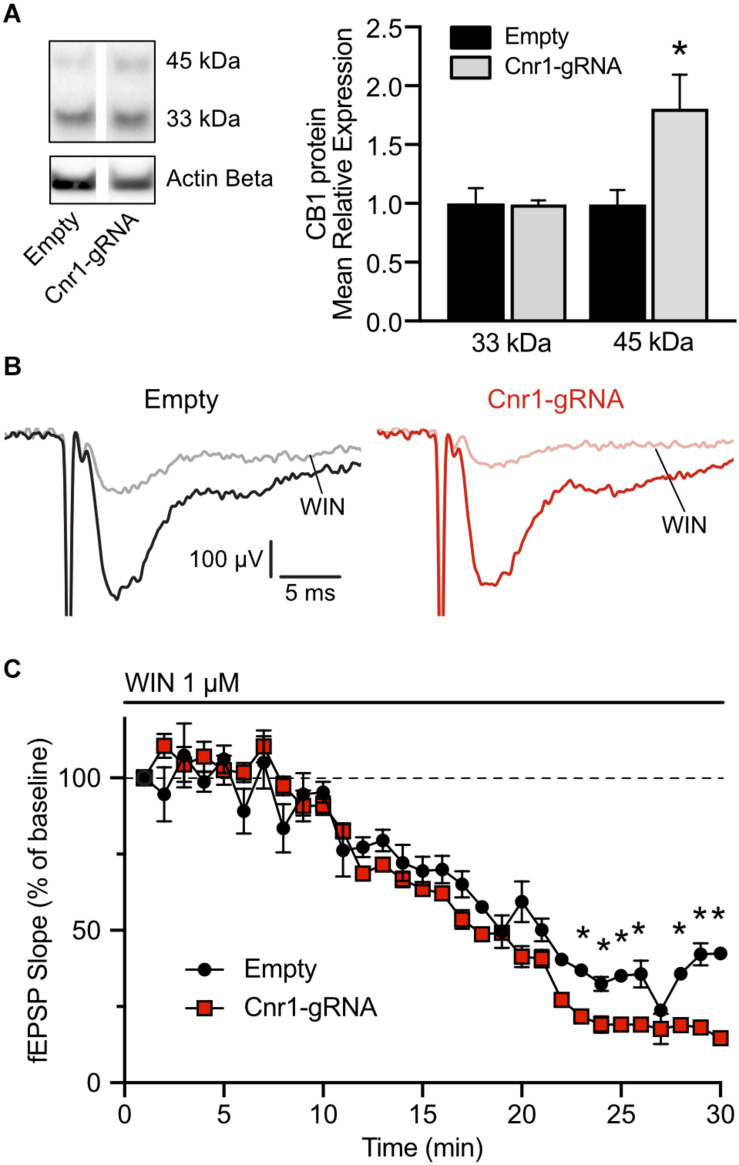
Expression of CB1 protein in the mouse hippocampus and functional validation of overexpression. **(A)** Total protein western blot from hippocampal tissue showing increased CB1 expression in animals treated with AAV-*Sa*dCas9-VPR and AAV-*Sa*dCas9-VPR (Cnr1-gRNA) compared to Empty vector controls (Cnr1-gRNA vs. Empty, Mann-Whitney test, *p < 0.05). **(B)** Representative traces showing fEPSPs recorded from CA1, before (black and dark red) and 25 min after WIN 55,212-22 application (WIN, gray and light red), in hippocampal slices from the Empty (left) and Cnr1-gRNA groups (right). Stimulation artifacts were cropped. **(C)** Quantification of fEPSP slope over time, displayed as percentage from baseline, during the period of WIN application (Cnr1-gRNA vs. Empty, Two-way repeated measures ANOVA followed by Sidak’s multiple comparison test, *p < 0.05).

The *in situ* hybridization data indicates that our AAV-based CRISPRa system can significantly upregulate *Cnr1* mRNA *in vivo*, but this does not necessarily translate into increased protein levels and does not offer any insight into the function of the upregulated gene. Toward these aims, we injected unilaterally another cohort of animals with AAV-*Sa*dCas9-VPR and AAV-Cnr1-gRNA or AAV-empty vector combinations, and 3–6 weeks later performed western blot and electrophysiological investigations to confirm CB1 upregulation and gain-of-function. After the slicing procedure for electrophysiology, some of the slices in the ipsilateral side were immediately frozen in dry ice, before proceeding with protein extraction and western blot analysis. Immunoblot quantification, similarly to what observed in primary cultures, showed two distinct bands and a significant upregulation of the 45 kDa band in slices animals injected with AAV-Cnr1-gRNA compared to AAV-empty (Empty vs. Cnr1-gRNA, FC 1.80 ± 0.28, *p* < 0.05, *n* = 6 animals) but no change in the 33 kDa band (Figure 8A).

The remaining hippocampal slices that were not processed for western blot were used for electrophysiological investigations of CB1-mediated decrease in synaptic transmission. Activation of CB1 receptors by endocannabinoids or other agonists induces downstream the inhibition of voltage-gated calcium channels and therefore reduces the release of neurotransmitters at the synapses on which it is expressed (Godino et al., 2007). A stimulating and a recording electrodes were placed in the stratum radiatum of area CA1, and field excitatory post-synaptic potentials (fEPSPs) were induced by electrical stimulation of Schaffer collaterals every 15 s. Representative traces are shown in Figure 8B. To quantify the CB1-mediated impact on synaptic transmission in these synapses, after measuring a 20 min baseline period, we applied in the bath the CB1 receptor agonist WIN 55,212-2 at a concentration of 1 μM and continuously monitored the pre-synaptic fiber volley and slope of the fEPSPs. In both slices from animals injected with AAV-*Sa*dCas9-VPR and AAV-Cnr1-gRNA (Cnr1-gRNA group) or AAV-*Sa*dCas9-VPR and AAV-empty (Empty group), we could observe a constant decrease in the slope amplitude starting from approximately 10 min after WIN application, peaking at 25 min. When comparing the relative decrease in slope over time between the two groups, we could observe that WIN had a stronger effect in the Cnr1-gRNA group starting from approximately 18 min after WIN application (Figure 8C, ^∗^*p* < 0.05, *n* = 6 slices from 6 animals per group), indicating that potentially an increased number of CB1 receptors at these synapses would induce a stronger reduction in pre-synaptic neurotransmitter release. Importantly, WIN effects in both groups were completely abolished by co-application of the CB1 receptor antagonist/inverse agonist AM251, and fiber volley amplitudes were constant over time (data not shown), confirming the specificity of this approach.

## Discussion

The study of complex neuronal function sometimes requires the use of tools able to specifically increase expression of target genes. In this study, we describe two flexible and efficient approaches for overexpressing target gene expression in neurons, and we demonstrate their ability to increase CB1 expression both *in vitro* and *in vivo*.

Classic gene overexpression approaches rely on the ability of lentiviruses or AAVs to deliver transgenes in target neurons, but are affected by several limitations. The packaging size of viral vectors in particular is limited, and does not allow the incorporation of long genes or combination of genes. These limitations could be largely overcome by using a CRISPRa system, where the transgene cargo size is fixed and gene targets are determined solely by altering sgRNAs sequences. Here, we packaged different variants of CRISPRa systems in both lentiviruses and AAVs and show that while both systems are similarly effective in overexpressing CB1 receptors *in vitro*, there is a significant difference in performance when injected in the brain *in vivo*.

Our first lentiviral based approach is based on a single-vector system where the “dead” Cas9 protein from *Streptococcus pyogenes (Sp)*, transcriptional activators and sgRNAs are packaged together in one virus. Using the EF1a promoter or CamKIIa promoter, this design only allows the insertion of smaller transcriptional activator modules, such as VP64, and not larger but theoretically more efficient fusion activators such as VPR (Chavez et al., 2015). To incorporate the VPR activator, and restrict expression the excitatory neurons, we developed a dual-vector system where expression of dCas9 and sgRNAs are confined to separate lentiviruses. Both systems were successful in inducing the expression of *Cnr1* mRNA and protein in MEFs and primary neuronal cultures, but surprisingly, we did not notice major differences in efficiency and gene induction strength between VP64 and VPR-based systems, as was instead previously described (Chavez et al., 2015; Bao et al., 2017), with both approaches able to increase expression by 3–4-fold in neurons. The amount of upregulation achieved by our system is similar to what previously observed by other research groups using both AAV and Lentivirus based approaches (Savell et al., 2019; Colasante et al., 2020), despite the use of different activator domains or target genes. This observation raises the interesting possibility that different activator domains might be more efficient in inducing expression of certain genes but not others, and even if multiple domains are used in concert to theoretically increase output, some genes cannot be overexpressed more than a given amount. In line with this concept, induction of *Cnr1* mRNA was significantly higher in MEFs (∼50-fold). This suggests, as previously described by others as well (Konermann et al., 2015; Chavez et al., 2016), that CRISPRa efficiency could be dependent on basal expression levels of the target genes, since MEFs do not normally express CB1 receptors while primary neurons physiologically do. In addition, at least for our AAV system based on *Sa*dCas9, multiple sgRNAs targeting the same gene at different locations are required to significantly increase mRNA transcription, as previously shown (Lau et al., 2019). Whether sgRNA synergy is required or not could, however, also be dependent on the specific gene targeted as in some cases one individual sgRNA is sufficient for significant gene induction of other typically neuronal genes (Colasante et al., 2020).

One notable observation from our western blot experiments was the presence of two discrete bands after immunodetection, one at approximately 30–35 kDa and the other at 45–50 kDa. CB1 receptors are glycosylated proteins, and different levels of glycosylation could explain the different apparent molecular weight observed on the membranes (Song and Howlett, 1995). In addition, CB1 mRNA could also undergo different splicing alternatives, resulting in an N-terminal modified isoform which is shorter than full length CB1 (Shire et al., 1995). However, this does not explain why our CRISPRa-induced overexpression only affected the larger molecular weight receptors, as increasing mRNA expression would theoretically result in proportional increase in protein levels from all alternative splicing forms, unless the splicing machinery would become overwhelmed and not able to process mRNAs as effectively when there are increased amounts present. This seems, however, unlikely as the mRNA increase was only 3–5-fold, but it’s a possibility that cannot be excluded. Alternatively, the presence of two bands could be explained by poor antibody performance and specificity, as it was only observed in western blots performed with the Immunogenes primary antibody and not with the Cayman antibody (which was used in Figure 5E). However, the Immunogenes antibody we used did not detect any protein in membranes loaded with samples from CB1 knock-out animal brains (data not shown), rendering this possible explanation highly unlikely. In addition, similar results have also been observed in other laboratories using the same primary antibody (Katona, unpublished observation), but the true nature of the two separate putative CB1 isoforms remains to be clearly elucidated.

To further extend the potential applications of our CRISPRa systems, we modified the dual vector approach for temporal control of target gene induction, by incorporating a Doxycycline sensitive induction control (TetON). In primary neurons transfected with the two lentiviruses, we were able to significantly induce CB1 expression by exposing cells to Dox for 1 or 6 days, and importantly, we did not detect significant induction of gene expression in the absence of Dox, validating the possibility that our CRISPRa dCas9-VPR system can be used when temporal control of gene induction is necessary. Similar systems have been used recently in the brain, for both CRISPRa and gene editing (Kumar et al., 2018; Colasante et al., 2020), indicating that such an approach could represent a robust strategy for temporal control of gene regulation.

To assess the functionality of the lentivirus-based dual vector system *in vivo*, we injected the viral combination in the hippocampus of wild type mice, and observed only a very limited expression of the reporter gene RFP in principal cell layers. Real time PCR analysis in the whole hippocampus revealed the expression of the dCas9 mRNA but failed to detect differences in *Cnr1* expression levels between groups. This was most likely due to the limited number of cells transduced by our viral combination, where non-transduced neurons masked the effect of a potential successful upregulation of *Cnr1* in transduced cells. The limited expression of transgene in the injected hippocampi, despite the injection of a combined 2 μL in four sites and a relatively high viral titer, is in line with what has been recently shown in other studies using similar CRISPRa approaches (Savell et al., 2019), and the reason has to be found on the intrinsic properties of the viral particles themselves. Lentiviruses have a medium-high packaging capacity but their larger size limits the viral spread from the injection sites, thereby resulting in a limited target area coverage. However, the use of lentiviruses could still be desirable for situations where the modification of only a limited number of neurons is necessary, as for the investigation of the impact of few cells on behavior (Zheng et al., 2018) or for interfering with small epileptic foci.

Our results with lentiviruses have prompted us to explore the possibility of applying an AAV-based CRISPRa approach to increase hippocampal coverage and thereby render the system more applicable for cases in which large areas need to be transduced. However, the *Sp*dCas9 protein fused to the VPR activator cannot be introduced into AAV particles due to the large size of their coding DNA, which exceeds the packaging capacity of AAVs. We therefore turned to CRISPRa system based on dCas9 from *Streptococcus aureus (Sa)*, a Cas9 ortholog with much smaller size and a smaller VPR domain (Ma et al., 2018). By modifying both the dCas9 protein and the VPR activator, we were able to fit the whole construct, including a neuron specific hSyn promoter, into AAV particles and produce viruses with high titer capable of transducing neurons both *in vitro* and *in vivo*.

*In vitro* performance of the AAV-based CRISPRa system was comparable to what we have seen with lentiviruses, but the coverage of hippocampal areas *in vivo* was significantly larger. With two injection sites and four total deposits of viral particles we were able to cover most of the dorso-ventral axis of the hippocampus. In addition, we demonstrate that using a dual viral vector approach does not present significant drawbacks, at least in the case of AAVs, as both *in vitro* and *in vivo* we observed very high co-transduction rates, indicating using two separate viral vectors to maximize expression of dCas9-VPR and sgRNAs separately can be used to activate genes as there is robust colocalization. However, when thinking about potential clinical applications of CRISPRa strategies, a dual vector system might not satisfy biosafety regulations for the use of AAVs in humans. In this case, continuous engineering of promoters, activator domains and regulatory sequences would be required to fit all components into a single AAV, in the cases where one sgRNA is sufficient to drive enough overexpression, as recently shown (Lau et al., 2019).

To quantify *Cnr1* mRNA expression in different hippocampal subregions we turned to RNAScope *in situ* hybridization, and observed a significant upregulation of *Cnr1* in the dentate gyrus and CA3 areas, but not in CA1. In particular, the upregulation was more pronounced in the DG, where basal expression levels of *Cnr1* are lower (Monory et al., 2006; Frazier, 2007), again confirming that CRISPRa tools are more efficient in inducing genes with low basal expression levels. In addition, western blot experiments confirmed that mRNA upregulation was reflected in protein expression, indicating that our system was functional *in vivo*, and could be used for interfering with CB1 expression in the hippocampus.

Nevertheless, increased CB1 protein levels at the western blot level does not necessarily mean that a gain-of-function can be achieved at the synaptic level, where CB1 receptors are normally expressed. It is possible that overexpressed receptors might not position correctly at synapses and could not be exerting their physiological function. It was previously shown, however, that overexpression of CB1 by AAV vectors was able to protect against seizure-induced excitotoxicity (Guggenhuber et al., 2010), indicating that overexpressed CB1 receptors should position and function correctly. To confirm the functionality of CB1 receptor overexpression, we performed field recordings in the CA1 area of the hippocampus from mice, and found that application of the CB1 agonist WIN 55,212-2 induced a bigger decrease in synaptic transmission in slices from animals injected with the vector combination containing *Cnr1* sgRNAs compared to those receiving the Empty vector. The observed decrease in synaptic transmission is in line with what previously observed by others in similar experimental setups (Kawamura et al., 2006; Takahashi and Castillo, 2006). Importantly, fiber volley amplitude was not affected, confirming that the effect was dependent on decrease in synaptic transmission and not on decreased afferent fiber stimulation over time.

The AAV-based system we describe here is made for constitutive expression, but could be easily adapted for temporal control of gene expression, using a similar approach as we described for our lentivirus-based CRISPRa vectors (Savell et al., 2019). In addition, it could be even made responsive to intrinsic neuronal activity, but incorporating activity-dependent promoters, or limited in expression to selective cell populations by adapting it for Cre-lox recombination strategies. These modifications will enable cell-type or circuit specific targeting to study the impact of target gene overexpression in more details.

The potential of CRISPRa gene therapy approaches for the treatment of neurological diseases is immense, also considering the relative ease of use and transferability to other models or even species. However, there are still several unknowns about the long-term use and safety of such approach. While it is certain that with time research will properly investigate this, at present the major drawback is represented by the uncertainty around the potential immunogenicity of bacterial proteins in human cells. It has been in fact previously shown that injection of AAVs encoding non-self-proteins (including the seemingly innocuous GFP) in non-human primates can elicit immune response and inflammation (Ciesielska et al., 2013; Yang et al., 2016), but whether that is the case for Cas9 and its derivates is presently unknown. Another concern revolves around the potential off-target effects of sgRNAs, but in this case the likelihood of deleterious effects is lower in CRISPRa approaches compared to CRISPR gene editing, as no DNA cleavage is involved.

## Conclusion

In conclusion, we show that gene modulation by CRISPRa *in vivo* in the brain can be successfully adapted to increase expression of synaptic proteins, and add to the growing CRISPRa toolbox for the potential development of gene therapy approaches against neurological disorders.

## Data Availability Statement

The original contributions presented in the study are included in the article/Supplementary Material, further inquiries can be directed to the corresponding author.

## Ethics Statement

The animal study was reviewed and approved by the Swedish Animal Welfare Agency.

## Author Contributions

VDM contributed to the design, performance and analysis of all the experiments. MR contributed to cloning lentiviral vectors. AB performed some Western Blot experiments. MM and LQ contributed to designing and performing the sgRNA validation. LQ contributed to designing the AAV-based *Sa*dCas9-VPR constructs. ML overseen all work, designed, performed, and analyzed the experiments. VDM and ML wrote the manuscript. All authors contributed to the article and approved the submitted version.

## Conflict of Interest

The authors declare that the research was conducted in the absence of any commercial or financial relationships that could be construed as a potential conflict of interest.

## Footnotes

1 https://chopchop.cbu.uib.no/

2 https://chopchop.cbu.uib.no
