# Supplementary figures and images for: Development and Validation of CRISPR Activator Systems for Overexpression of CB1 Receptors in Neurons
Source: Front Mol Neurosci. 2020 Sep 8;13:168. doi: 10.3389/fnmol.2020.00168 (PMC7506083; doi:10.3389/fnmol.2020.00168)

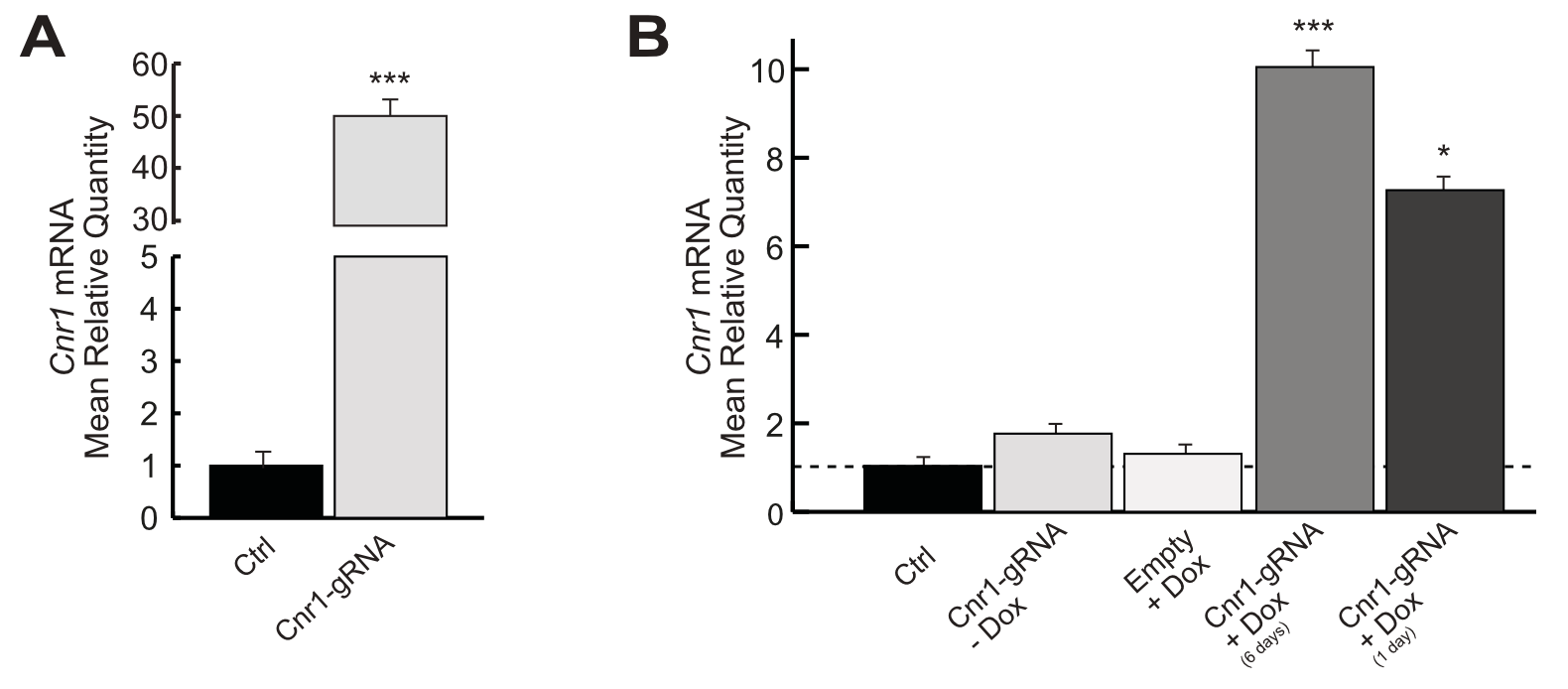

Supplement: Supplementary file 1 [file Image_1.TIFF]

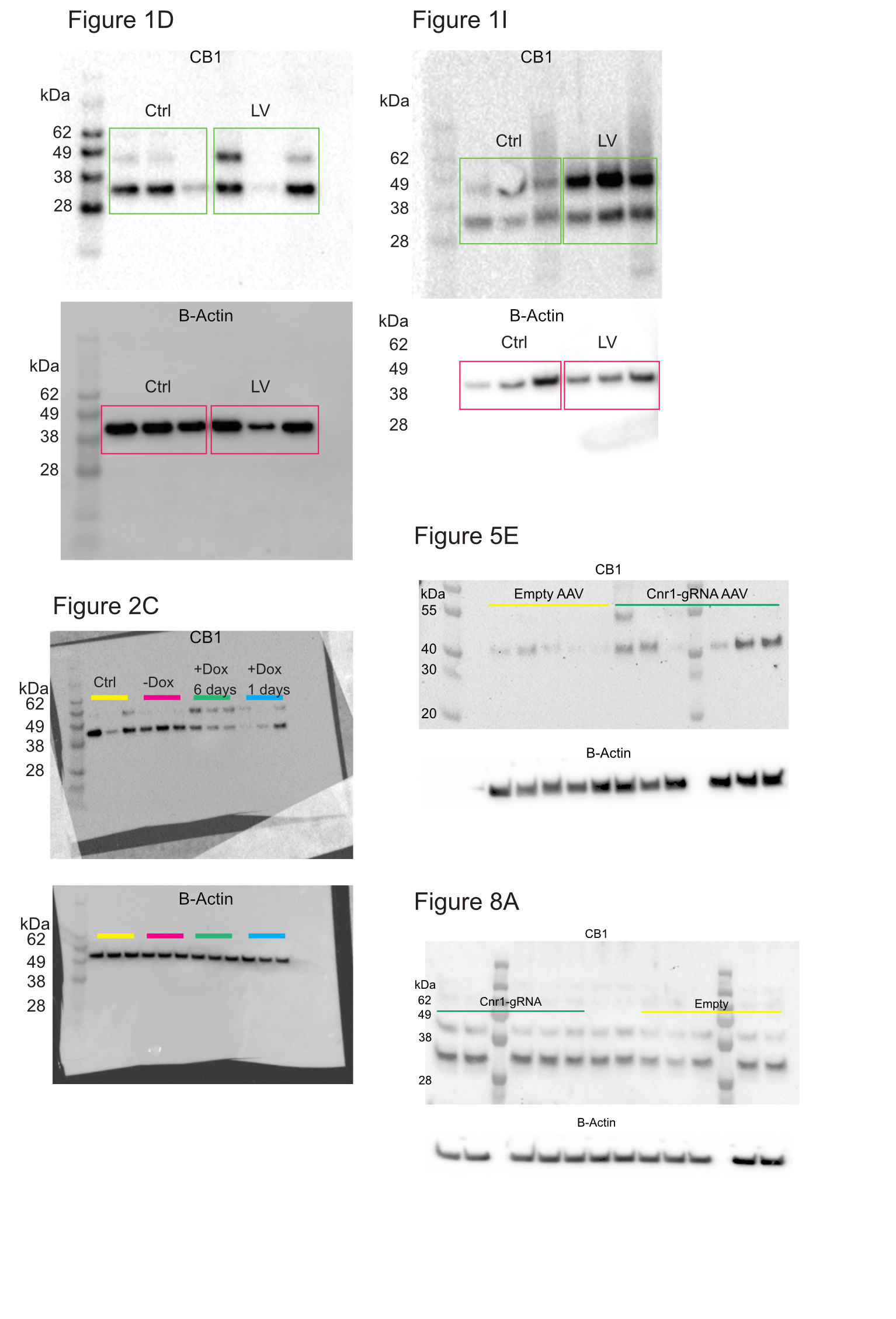

Supplement: Supplementary file 2 [file Image_2.TIFF]
