# Supplementary material for: Development and Validation of CRISPR Activator Systems for Overexpression of CB1 Receptors in Neurons
Source: Front Mol Neurosci. 2020 Sep 8;13:168. doi: 10.3389/fnmol.2020.00168 (PMC7506083; doi:10.3389/fnmol.2020.00168)
Supplement: Supplementary file 3 [file Data_Sheet_1.docx]

**Supplementary Data**

**Supplementary Figure 1. Cnr1 expression in Mouse Embryonic Fibroblast (MEF). A.** The bar graphs show the mRNA expression level of the *Cnr1* gene in MEF infected with single lentiviral vector LV-*Sp*dCas9-VP64-Cnr1 (Cnr1-gRNA) compared with non-treated controls (Ctrl). The graph represents the results of RT-PCR obtained using the ΔΔCt method to calculate the Mean Relative Quantity of the tested genes. The values are given as mean ± SEM (unpaired t-test, ****p*<0.0001, n=6). **B.** The bar graphs show the mRNA expression level of the *Cnr1* gene in MEF infected with the DoxCRISPR-Cas9 dual lentiviral system at two different time points (Cnr1-gRNA + Dox, 1 and 6 days after exposure to Dox), compared to non-infected (Ctrl), non-treated (Cnr1-gRNA - Dox) controls and cells infected with an empty gRNA vector and exposed to Dox (Empty + Dox). The graphs represent the results of RT-PCR obtained using the ΔΔCt method to calculate the Mean Relative Quantity of the tested genes. The values are given as mean ± SEM (One-way ANOVA followed by Dunnet’s multiple comparison test, **p*<0.05; ****p*<0.0001, n=6).

**Supplementary Table 1.** sgRNA sequences used in this study

| **Vector** | **gRNA** | **Sequence** | **Distance from Cnr1 TSS** | **Promoter** |
| --- | --- | --- | --- | --- |
| LV | 1 | CCCCCGGGCCAGCGCCGCGG | 174 | mU6 |
| LV | 2 | GCTGCCGCCGCTGCCAGGGC | 74 | hU6 |
| LV | 3 | GGACCGGCGCGCGGCGCCCG | 105 | hH1 |
| LV | 4 | CCAGTCCCATTTATGAAGCG | 2 | h7SK |
| AAV | 1 | GCCTTCCTGCCGCCCTGTTT | 418 | mU6 |
| AAV | 2 | GAGGGCGCCAGGGAGCAGAG | 275 | hU6 |
| AAV | 3 | GGTCGTTGGTGGCAAAGAGT | 239 | hH1 |
| AAV | 4 | TCCCCGCGCAGATCCCTTGG | 104 | h7SK |

**Supplementary Table 2.** Titer of the Lentivirus and AAV used in this study

| **Lentivirus** | **Titer** |
| --- | --- |
| LV-*Sp*dCas9-VP64-Cnr1 | 3,4 x 10^8^ |
| LV-*Sp*dCas9-VPR | 1,4 x 10^8^ |
| LV-Cnr1-gRNA | 2,2 x 10^8^ |
| LV-Cnr1-gRNA-rtTA-GFP | 4,6 x 10^8^ |
| LV-TRE-*Sp*dCas9-VPR | 1,2 x 10^9^ |
| **AAV** | **Titer** |
| AAV-Empty | 9,9 x 10^12^ |
| AAV-*Sa*dCas9-VPR | 7,6 x 10^12^ |
| AAV-Cnr1-gRNA | 6,7 x 10^12^ |

**Supplementary Table 3.** RNAscope probes, target regions and number of pairs for the in situ hybridization experiments described in this study

| **RNAscope® Probe** | **Target Region** | **Number of pairs** |
| --- | --- | --- |
| Mm-Cnr1, 420721 | 530 - 1458 | 20 |
| Mn-saCas9 501621 | 699 - 1732 | 20 |
| Mm-Ppib 313911 | 98 - 856 | 15 |
| Mm-DapB 310043 | 414 - 862 | 10 |
| GFP | 628 - 1352 | 13 |

**Supplementary Table 4.** Full list of potential off-target sites from all four sgRNAs packaged in the AAV-sgRNA-Cnr1 vector

| crRNA | DNA | Chromosome | Position | Direction | Mismatches |
| --- | --- | --- | --- | --- | --- |
| GCCTTCCTGCCGCCCTGTTTNNGRRT | tCCTTCCTtCCctCCTGTTTTTGAGT | chr1 | 14888672 | - | 4 |
| GAGGGCGCCAGGGAGCAGAGNNGRRT | GtGGGaGCtAGGGAGCAGgGGTGGGT | chr1 | 36092974 | + | 4 |
| GAGGGCGCCAGGGAGCAGAGNNGRRT | GAGGGCGCCAaGcAGaAGAGTTGAGT | chr1 | 57770017 | + | 3 |
| GCCTTCCTGCCGCCCTGTTTNNGRRT | GCCTggCTGCaGCCCTGcTTCGGGAT | chr1 | 65311277 | + | 4 |
| GAGGGCGCCAGGGAGCAGAGNNGRRT | GtatGtGCCAGGGAGCAGAGCTGGAT | chr1 | 83868082 | - | 4 |
| GAGGGCGCCAGGGAGCAGAGNNGRRT | aAGGGgtCCtGGGAGCAGAGGGGGAT | chr1 | 94552873 | + | 4 |
| GGTCGTTGGTGGCAAAGAGTNNGRRT | GGcCaTTGGTGGCAAccAGTGGGAGT | chr1 | 105005820 | + | 4 |
| GCCTTCCTGCCGCCCTGTTTNNGRRT | GCCcTCCTcCCtCCaTGTTTTAGAGT | chr1 | 120127918 | + | 4 |
| GAGGGCGCCAGGGAGCAGAGNNGRRT | GAGGatGCCAGGGAGgtGAGGTGGGT | chr1 | 140529048 | - | 4 |
| GGTCGTTGGTGGCAAAGAGTNNGRRT | aGTgGTgtGTGGCAAAGAGTAGGGAT | chr1 | 168028761 | + | 4 |
| GAGGGCGCCAGGGAGCAGAGNNGRRT | GgGGGgGgCAGGGAGCgGAGGTGAGT | chr1 | 180554180 | + | 4 |
| GAGGGCGCCAGGGAGCAGAGNNGRRT | GAGGGgaCCAGGaAGCAGAGATGGAT | chr1 | 186165702 | + | 3 |
| GAGGGCGCCAGGGAGCAGAGNNGRRT | GAGtGCGggAGGGAGgAGAGCAGAGT | chr10 | 7703923 | + | 4 |
| GCCTTCCTGCCGCCCTGTTTNNGRRT | GCCTgCCTGCCtgCCTGTcTATGGAT | chr10 | 11836050 | + | 4 |
| GAGGGCGCCAGGGAGCAGAGNNGRRT | tAGGGgGtCAGGGAGCAGgGGAGGGT | chr10 | 40497053 | + | 4 |
| GAGGGCGCCAGGGAGCAGAGNNGRRT | GgGGGCGCgAGGtAGCtGAGGAGGGT | chr10 | 42842545 | + | 4 |
| GCCTTCCTGCCGCCCTGTTTNNGRRT | GCCTTtCTGCCtCCCTGTgcTAGGGT | chr10 | 43710873 | - | 4 |
| GAGGGCGCCAGGGAGCAGAGNNGRRT | cAGGGaaCCAGGGAGaAGAGCAGAGT | chr10 | 51965835 | - | 4 |
| GGTCGTTGGTGGCAAAGAGTNNGRRT | ttTCaTgGGTGGCAAAGAGTGTGGAT | chr10 | 60153059 | + | 4 |
| GAGGGCGCCAGGGAGCAGAGNNGRRT | GAGGGCagCAGtGAGCAGgGAAGGGT | chr10 | 61428748 | + | 4 |
| GAGGGCGCCAGGGAGCAGAGNNGRRT | agGGGgGCCAGGGgGCAGAGCAGAAT | chr10 | 72097103 | - | 4 |
| GAGGGCGCCAGGGAGCAGAGNNGRRT | GAGGGtGCCgGGGtGCAGgGGCGGGT | chr10 | 81482374 | + | 4 |
| TCCCCGCGCAGATCCCTTGGNNGRRT | TCCCaGCaCAGATCtCTgGGTAGAAT | chr10 | 85621376 | - | 4 |
| GGTCGTTGGTGGCAAAGAGTNNGRRT | GGTCtgTGGTGcCcAAGAGTCAGAGT | chr10 | 109227423 | + | 4 |
| TCCCCGCGCAGATCCCTTGGNNGRRT | TCCCtGtGCAGAgCCCTTcGAGGGAT | chr10 | 117284690 | + | 4 |
| GCCTTCCTGCCGCCCTGTTTNNGRRT | GaggTCCTGCaGCCCTGTTTCAGGAT | chr10 | 123274579 | - | 4 |
| GCCTTCCTGCCGCCCTGTTTNNGRRT | GCCTTgCTcCCtCCCTGTTgATGGGT | chr10 | 127464847 | + | 4 |
| GAGGGCGCCAGGGAGCAGAGNNGRRT | aAGGGaGCaAGaGAGCAGAGCTGAAT | chr11 | 45538414 | - | 4 |
| GCCTTCCTGCCGCCCTGTTTNNGRRT | aCCTgaCTGCCaCCCTGTTTCAGAAT | chr11 | 50546610 | - | 4 |
| GAGGGCGCCAGGGAGCAGAGNNGRRT | GAGaGgGCCAGGGAGgAGAaCGGAGT | chr11 | 58341496 | - | 4 |
| GAGGGCGCCAGGGAGCAGAGNNGRRT | GAaGGCcCCAGGGtGCAGtGGTGAGT | chr11 | 60729656 | - | 4 |
| GAGGGCGCCAGGGAGCAGAGNNGRRT | GAGaGgGCCAGGGAGggGAGGCGGAT | chr11 | 61024136 | + | 4 |
| GAGGGCGCCAGGGAGCAGAGNNGRRT | GgGGGCGCCAGGGtaCAGAtAAGAAT | chr11 | 61270291 | + | 4 |
| GAGGGCGCCAGGGAGCAGAGNNGRRT | GAGGagGCCAGGGctCAGAGCAGGGT | chr11 | 78349019 | - | 4 |
| GGTCGTTGGTGGCAAAGAGTNNGRRT | GGTgGTgtGTGGCAAAGAGaAGGGGT | chr11 | 82477517 | - | 4 |
| GAGGGCGCCAGGGAGCAGAGNNGRRT | GAGGGCGCCAGGaAcCAaAcCTGGAT | chr11 | 97666347 | - | 4 |
| GCCTTCCTGCCGCCCTGTTTNNGRRT | cCCTaCCTGCaGCCCTGTTgCAGAGT | chr11 | 102207333 | - | 4 |
| GAGGGCGCCAGGGAGCAGAGNNGRRT | GAGGGtGCCAGGGAGCAttcATGGGT | chr12 | 28590868 | - | 4 |
| GAGGGCGCCAGGGAGCAGAGNNGRRT | GAGtGCaCCAGGcAGCtGAGGAGAGT | chr12 | 41645045 | + | 4 |
| GCCTTCCTGCCGCCCTGTTTNNGRRT | GCCTTCCTcCCGCCCaGTgcTGGGAT | chr12 | 55839209 | + | 4 |
| GAGGGCGCCAGGGAGCAGAGNNGRRT | GAGGGCctCtGGGAGaAGAGTGGAAT | chr12 | 117678872 | + | 4 |
| GCCTTCCTGCCGCCCTGTTTNNGRRT | GCCTgCCTGCCtCCCTGTgcTGGAAT | chr13 | 9250083 | - | 4 |
| GCCTTCCTGCCGCCCTGTTTNNGRRT | GCCTTCCTGCatCtCTGcTTCCGGAT | chr13 | 25172035 | + | 4 |
| GAGGGCGCCAGGGAGCAGAGNNGRRT | GAGGcCaCCAGGGAGCAcAaCAGAAT | chr13 | 34148459 | - | 4 |
| GAGGGCGCCAGGGAGCAGAGNNGRRT | cAaGGgGCCAGGGAGaAGAGTGGGAT | chr13 | 72721459 | + | 4 |
| GGTCGTTGGTGGCAAAGAGTNNGRRT | GagCcTTGGTGGCAtAGAGTTAGAAT | chr13 | 79704295 | - | 4 |
| GAGGGCGCCAGGGAGCAGAGNNGRRT | GAGGaaGCCAGGGcaCAGAGGTGAAT | chr13 | 81221199 | + | 4 |
| GAGGGCGCCAGGGAGCAGAGNNGRRT | GAGGaaGCCAGaGAGCAGAGAGGAAT | chr13 | 83497923 | - | 3 |
| GCCTTCCTGCCGCCCTGTTTNNGRRT | tCCTTCgTtCCGCCCTGaTTCTGGGT | chr13 | 95880491 | - | 4 |
| GGTCGTTGGTGGCAAAGAGTNNGRRT | GGTCtTatGTGGCcAAGAGTTAGGGT | chr14 | 22382923 | + | 4 |
| GCCTTCCTGCCGCCCTGTTTNNGRRT | tCCTTCCTGCCtCaCTGcTTCTGGAT | chr14 | 30636520 | + | 4 |
| GAGGGCGCCAGGGAGCAGAGNNGRRT | GAGGGgGCtgGGGAGgAGAGGGGAAT | chr14 | 34141331 | + | 4 |
| GCCTTCCTGCCGCCCTGTTTNNGRRT | GCCTTCCTGCCattCTcTTTCTGAGT | chr14 | 45649209 | - | 4 |
| GAGGGCGCCAGGGAGCAGAGNNGRRT | GtGGGtGCCAGGGAtCAaAGTTGGAT | chr14 | 47970407 | + | 4 |
| GCCTTCCTGCCGCCCTGTTTNNGRRT | GCCTTCCTGCCtCtCTGTTTGTGGAT | chr14 | 75433514 | + | 2 |
| GCCTTCCTGCCGCCCTGTTTNNGRRT | GgCTTCCgGCtGtCCTGTTTATGAAT | chr14 | 99408890 | - | 4 |
| GAGGGCGCCAGGGAGCAGAGNNGRRT | GAGGGCtCCtGGaAGgAGAGGAGAGT | chr15 | 19461301 | - | 4 |
| GCCTTCCTGCCGCCCTGTTTNNGRRT | GCCTgCCTGCCtgCCTGTcTCAGAGT | chr15 | 72209459 | + | 4 |
| GCCTTCCTGCCGCCCTGTTTNNGRRT | GCCTTCtTaCCtCtCTGTTTTGGGGT | chr15 | 77028098 | + | 4 |
| GAGGGCGCCAGGGAGCAGAGNNGRRT | GAGGGaGCCAGaGAGgAaAGAGGGAT | chr15 | 78520536 | - | 4 |
| GAGGGCGCCAGGGAGCAGAGNNGRRT | GAGGtCtCCAGtGAGCAGgGATGAGT | chr15 | 97564548 | - | 4 |
| GAGGGCGCCAGGGAGCAGAGNNGRRT | GAGtGCtaCAGGGgGCAGAGCTGGAT | chr15 | 98137801 | - | 4 |
| GAGGGCGCCAGGGAGCAGAGNNGRRT | GAGGGCcCaAGGGAGgAtAGTTGAAT | chr16 | 8135388 | + | 4 |
| GAGGGCGCCAGGGAGCAGAGNNGRRT | GAGGGCtCCAGGGAGCcGAaAGGGGT | chr16 | 17489803 | - | 3 |
| GGTCGTTGGTGGCAAAGAGTNNGRRT | GGgtGTTGGTGGCAcAGAGgCGGGGT | chr16 | 18703814 | + | 4 |
| GAGGGCGCCAGGGAGCAGAGNNGRRT | GAGGGCGgCgGcGAGCgGAGCGGGGT | chr16 | 33684543 | - | 4 |
| GGTCGTTGGTGGCAAAGAGTNNGRRT | GtTtGTTGGTGtCAcAGAGTAGGAAT | chr16 | 66207224 | + | 4 |
| GCCTTCCTGCCGCCCTGTTTNNGRRT | GCCTgCCTGCCtgCCTGTcTCTGAGT | chr16 | 89729353 | - | 4 |
| GCCTTCCTGCCGCCCTGTTTNNGRRT | tCCTTCCTGtgGCCCaGTTTAGGAAT | chr16 | 92056737 | - | 4 |
| GAGGGCGCCAGGGAGCAGAGNNGRRT | GAaGGCGCagGGGAGCAGgGGAGGAT | chr16 | 93438636 | + | 4 |
| GAGGGCGCCAGGGAGCAGAGNNGRRT | GAGGGaGggAGGGAGgAGAGATGGAT | chr17 | 4739439 | - | 4 |
| TCCCCGCGCAGATCCCTTGGNNGRRT | TCCCaGCtCAGATCCCagGGAAGGGT | chr17 | 31637145 | + | 4 |
| GGTCGTTGGTGGCAAAGAGTNNGRRT | GGaCagTGaTGGCAAAGAGTAAGGGT | chr17 | 32520470 | - | 4 |
| GCCTTCCTGCCGCCCTGTTTNNGRRT | GgCTTCCTGCtGCCaaGTTTGTGGGT | chr17 | 47269384 | + | 4 |
| GAGGGCGCCAGGGAGCAGAGNNGRRT | GAGGGCtCttGGGAGCAGtGGGGAGT | chr17 | 84029985 | - | 4 |
| TCCCCGCGCAGATCCCTTGGNNGRRT | TCtaCGtGCAGATCCCTTGGCCGGAT | chr18 | 11258872 | + | 3 |
| GCCTTCCTGCCGCCCTGTTTNNGRRT | GCCTgCCTGCCtCCaTGaTTCTGAAT | chr18 | 44306965 | + | 4 |
| TCCCCGCGCAGATCCCTTGGNNGRRT | aCCCCGgtCAGATCCCcTGGGGGGAT | chr18 | 53625114 | + | 4 |
| GAGGGCGCCAGGGAGCAGAGNNGRRT | cAGGtCcCCAGGGtGCAGAGTGGGGT | chr18 | 60628636 | - | 4 |
| GGTCGTTGGTGGCAAAGAGTNNGRRT | GGTCGaTGGTGaCAAAGAGgAGGAGT | chr18 | 63050457 | - | 3 |
| TCCCCGCGCAGATCCCTTGGNNGRRT | TCCCaGCGtAGAgCCCTgGGCTGAAT | chr18 | 74730274 | + | 4 |
| GAGGGCGCCAGGGAGCAGAGNNGRRT | agGGGaGaCAGGGAGCAGAGACGGGT | chr18 | 77511001 | - | 4 |
| GAGGGCGCCAGGGAGCAGAGNNGRRT | tAGGGgcCCAGGGAGaAGAGGAGGGT | chr19 | 42207389 | - | 4 |
| GAGGGCGCCAGGGAGCAGAGNNGRRT | GAGGGttgCAGGcAGCAGAGATGAGT | chr19 | 58595125 | + | 4 |
| GAGGGCGCCAGGGAGCAGAGNNGRRT | GAGGGgGaCAGGtAaCAGAGGAGGAT | chr2 | 4333162 | + | 4 |
| GAGGGCGCCAGGGAGCAGAGNNGRRT | GAGGGaGaCAGtGAGCtGAGCAGGGT | chr2 | 9656599 | + | 4 |
| GGTCGTTGGTGGCAAAGAGTNNGRRT | GGTtGTTaGTGGCtAAGAGaCAGGAT | chr2 | 19372272 | + | 4 |
| GAGGGCGCCAGGGAGCAGAGNNGRRT | GAGGGaGaCtGGGAGgAGAGGGGGGT | chr2 | 22894708 | + | 4 |
| TCCCCGCGCAGATCCCTTGGNNGRRT | TCCCCaCGCAcAgtCCTTGGAAGGGT | chr2 | 27187918 | - | 4 |
| GAGGGCGCCAGGGAGCAGAGNNGRRT | GAGGcCctCAGGGAGCcGAGAGGAGT | chr2 | 27713213 | + | 4 |
| GAGGGCGCCAGGGAGCAGAGNNGRRT | GttGGtcCCAGGGAGCAGAGTTGGGT | chr2 | 28553748 | - | 4 |
| GAGGGCGCCAGGGAGCAGAGNNGRRT | GAGGGaGaCtGGGAGgAGAGATGGGT | chr2 | 35568910 | + | 4 |
| GAGGGCGCCAGGGAGCAGAGNNGRRT | GAGGGtGgCAGGaAaCAGAGAAGGAT | chr2 | 93161724 | - | 4 |
| GAGGGCGCCAGGGAGCAGAGNNGRRT | GAGGGCtaCAtGGAGCtGAGTGGGGT | chr2 | 117198091 | - | 4 |
| GCCTTCCTGCCGCCCTGTTTNNGRRT | tCCTTCCTaCaGCCCTcTTTCTGAGT | chr2 | 167819585 | + | 4 |
| GAGGGCGCCAGGGAGCAGAGNNGRRT | GAGGGCtCCtaGGAGCAGtGAAGGGT | chr3 | 121201051 | - | 4 |
| GCCTTCCTGCCGCCCTGTTTNNGRRT | cCCTTCCTcCCtCCaTGTTTGTGGGT | chr4 | 5067119 | - | 4 |
| GAGGGCGCCAGGGAGCAGAGNNGRRT | GAcGGCGgCgGGGAGCAGgGTCGGAT | chr4 | 32983738 | + | 4 |
| GCCTTCCTGCCGCCCTGTTTNNGRRT | GCCTTCCTGCCGCCCTGTTTCCGGAT | chr4 | 33924212 | + | 0 |
| GAGGGCGCCAGGGAGCAGAGNNGRRT | GAGGGCGCCAGGGAGCAGAGCAGGGT | chr4 | 33924355 | + | 0 |
| GGTCGTTGGTGGCAAAGAGTNNGRRT | GGTCGTTGGTGGCAAAGAGTGAGGAT | chr4 | 33924391 | + | 0 |
| TCCCCGCGCAGATCCCTTGGNNGRRT | TCCCCGCGCAGATCCCTTGGCGGAGT | chr4 | 33924526 | + | 0 |
| GAGGGCGCCAGGGAGCAGAGNNGRRT | GAtGGgGCCAGGaAGCAGAaGTGGGT | chr4 | 93975759 | + | 4 |
| GCCTTCCTGCCGCCCTGTTTNNGRRT | GCtTTCCTGCtcCCCTGTTgCTGAAT | chr4 | 95058673 | - | 4 |
| GCCTTCCTGCCGCCCTGTTTNNGRRT | GCCTgCCTGCCtgCCTGTcTGTGGAT | chr4 | 97186724 | - | 4 |
| GGTCGTTGGTGGCAAAGAGTNNGRRT | GGaCaTgGGaGGCAAAGAGTGGGGGT | chr4 | 106104376 | - | 4 |
| GCCTTCCTGCCGCCCTGTTTNNGRRT | GtCTTCCTGgCcCCCTtTTTCAGAGT | chr4 | 116334192 | + | 4 |
| GAGGGCGCCAGGGAGCAGAGNNGRRT | GctGGCaCCAGGGAGCAGtGCTGGGT | chr4 | 117254782 | + | 4 |
| GAGGGCGCCAGGGAGCAGAGNNGRRT | GAGGGaGggAGGGAGgAGAGATGGAT | chr4 | 120156007 | + | 4 |
| GAGGGCGCCAGGGAGCAGAGNNGRRT | GAGGGCtaCAGaGAGCAGAtGAGGAT | chr4 | 134711603 | + | 4 |
| GCCTTCCTGCCGCCCTGTTTNNGRRT | cCCTTCCTGCCcCtCTtTTTAAGGGT | chr4 | 141940124 | - | 4 |
| GCCTTCCTGCCGCCCTGTTTNNGRRT | aCCTTCCTGCCtCtCTGTTcTGGGGT | chr4 | 142178815 | + | 4 |
| GAGGGCGCCAGGGAGCAGAGNNGRRT | GtGGGgGgCAGGcAGCAGAGGGGAGT | chr5 | 19832103 | + | 4 |
| GAGGGCGCCAGGGAGCAGAGNNGRRT | GgGGagGCCtGGGAGCAGAGCTGGGT | chr5 | 27711316 | - | 4 |
| GAGGGCGCCAGGGAGCAGAGNNGRRT | GAGGGgGtgAGtGAGCAGAGATGGGT | chr5 | 30995973 | + | 4 |
| GAGGGCGCCAGGGAGCAGAGNNGRRT | GAGGGgGtCAGGGAGatGAGTGGGAT | chr5 | 51671538 | + | 4 |
| GAGGGCGCCAGGGAGCAGAGNNGRRT | GAtGGCaCCAGGaAGCAaAGTTGAAT | chr5 | 89106263 | + | 4 |
| GGTCGTTGGTGGCAAAGAGTNNGRRT | GGTCcTTGGTGGaAAAcAGgAAGGAT | chr5 | 110344029 | - | 4 |
| GCCTTCCTGCCGCCCTGTTTNNGRRT | GCCTgCCTGCCtgCCTGTgTGAGGGT | chr5 | 120411886 | - | 4 |
| GAGGGCGCCAGGGAGCAGAGNNGRRT | GgtGGCGgtAGGGAGCAGAGCAGGAT | chr5 | 125324351 | + | 4 |
| GAGGGCGCCAGGGAGCAGAGNNGRRT | GAGGGCtCCtGGGAGCtGgGATGGAT | chr5 | 127488338 | - | 4 |
| GAGGGCGCCAGGGAGCAGAGNNGRRT | GAaGaaGCCAaGGAGCAGAGAGGAAT | chr5 | 130783745 | + | 4 |
| GAGGGCGCCAGGGAGCAGAGNNGRRT | GAGGGgcCCAGtGAGgAGAGGTGGGT | chr5 | 135874871 | + | 4 |
| GAGGGCGCCAGGGAGCAGAGNNGRRT | GgGGGgagCAGGGAGCAGAGCTGAAT | chr6 | 4838485 | + | 4 |
| GAGGGCGCCAGGGAGCAGAGNNGRRT | GgaGGCcCCAGGGAGaAGAGACGAGT | chr6 | 6548282 | - | 4 |
| GCCTTCCTGCCGCCCTGTTTNNGRRT | GCCTgCCTGCCcgCCTGcTTGTGGAT | chr6 | 28379160 | + | 4 |
| GGTCGTTGGTGGCAAAGAGTNNGRRT | GGaaGTgtGTGGCAAAGAGTAGGGGT | chr6 | 34607931 | - | 4 |
| GAGGGCGCCAGGGAGCAGAGNNGRRT | GAGGGaGggAGGGAGgAGAGAGGAGT | chr6 | 82584123 | + | 4 |
| GCCTTCCTGCCGCCCTGTTTNNGRRT | GCCTTCaTGggGCaCTGTTTAGGGGT | chr6 | 87100218 | + | 4 |
| GCCTTCCTGCCGCCCTGTTTNNGRRT | GCCTaCCTGCaGCtCTGTgTGTGAAT | chr6 | 89423089 | + | 4 |
| GGTCGTTGGTGGCAAAGAGTNNGRRT | GGTgGTTGGgaGCAgAGAGTTTGAGT | chr6 | 98975016 | - | 4 |
| GAGGGCGCCAGGGAGCAGAGNNGRRT | tAGaGCGCCAtGGAGCAGtGATGGGT | chr6 | 122843465 | + | 4 |
| GAGGGCGCCAGGGAGCAGAGNNGRRT | GAGtGCGgCgaGGAGCAGAGTAGGGT | chr6 | 125593574 | - | 4 |
| GCCTTCCTGCCGCCCTGTTTNNGRRT | GCCTgCCTGCtGCCaTGTTaCTGGAT | chr6 | 140919696 | - | 4 |
| GAGGGCGCCAGGGAGCAGAGNNGRRT | GAGGGtcCtAGGGAGCAGAGAAGAAT | chr7 | 3310544 | - | 3 |
| GCCTTCCTGCCGCCCTGTTTNNGRRT | GCCTgCCTGCCtgCCTGTaTGCGGGT | chr7 | 4901435 | - | 4 |
| GCCTTCCTGCCGCCCTGTTTNNGRRT | tCCTTCCTtCCtCCCTtTTTTGGGGT | chr7 | 28552083 | - | 4 |
| GAGGGCGCCAGGGAGCAGAGNNGRRT | aAGGaaGCCAGGaAGCAGAGAAGAAT | chr7 | 30988118 | - | 4 |
| GGTCGTTGGTGGCAAAGAGTNNGRRT | GGTCGTgGGgGGCAAgGtGTGAGGAT | chr7 | 34415343 | - | 4 |
| GCCTTCCTGCCGCCCTGTTTNNGRRT | GCCTTCaTGCaGCCCTGTTTATGGAT | chr7 | 37842316 | - | 2 |
| GAGGGCGCCAGGGAGCAGAGNNGRRT | GAGGGgaCCAGGGAGCtGgGGGGAGT | chr7 | 73710481 | - | 4 |
| TCCCCGCGCAGATCCCTTGGNNGRRT | TCCCCaCGCAGgTCtCTTtGGGGAAT | chr7 | 75287383 | - | 4 |
| GAGGGCGCCAGGGAGCAGAGNNGRRT | cAGcGCGCCtGGGAGaAGAGCCGGGT | chr7 | 80339943 | + | 4 |
| GCCTTCCTGCCGCCCTGTTTNNGRRT | tCCTcCCTGCCtCCCTtTTTGAGAAT | chr7 | 121039742 | + | 4 |
| GCCTTCCTGCCGCCCTGTTTNNGRRT | tCCTTCCTcCacCCCTGTTTCTGAAT | chr7 | 121418108 | + | 4 |
| GAGGGCGCCAGGGAGCAGAGNNGRRT | GAGtGCtaCAGGGAGaAGAGTGGAGT | chr7 | 134351664 | - | 4 |
| GAGGGCGCCAGGGAGCAGAGNNGRRT | GAGGGCcCCAGGaAGgAGtGGTGGAT | chr7 | 144851870 | + | 4 |
| GCCTTCCTGCCGCCCTGTTTNNGRRT | cCCTTCCctCCcCCCTGTTTCGGGGT | chr8 | 15332270 | - | 4 |
| GAGGGCGCCAGGGAGCAGAGNNGRRT | GAGGGCtCaAGGGAGgAcAGTTGAAT | chr8 | 64142052 | + | 4 |
| GCCTTCCTGCCGCCCTGTTTNNGRRT | GCtTTCCTGCaGtCCaGTTTAAGGGT | chr8 | 87568142 | + | 4 |
| GAGGGCGCCAGGGAGCAGAGNNGRRT | GAGcGgGCCAtGGAGCAaAGGTGGGT | chr8 | 100059987 | + | 4 |
| GAGGGCGCCAGGGAGCAGAGNNGRRT | cAGGcCaCCAGGaAGCAGAGGTGAGT | chr8 | 117280188 | + | 4 |
| GAGGGCGCCAGGGAGCAGAGNNGRRT | GAGGGCagCgGGaAGCAGAGCTGGGT | chr8 | 118468751 | - | 4 |
| GAGGGCGCCAGGGAGCAGAGNNGRRT | GAGGGaGaCtGGGAGaAGAGATGAGT | chr8 | 120616955 | + | 4 |
| GAGGGCGCCAGGGAGCAGAGNNGRRT | GAGGGCcCaAGGGAGCAGgGAGGGGT | chr8 | 122204732 | + | 3 |
| GCCTTCCTGCCGCCCTGTTTNNGRRT | GCCTgCCTGCCtgCCTGTcTGTGGAT | chr8 | 126883008 | + | 4 |
| GAGGGCGCCAGGGAGCAGAGNNGRRT | GAGGaaGCCAGaGAGtAGAGAAGAGT | chr9 | 4930599 | + | 4 |
| GAGGGCGCCAGGGAGCAGAGNNGRRT | GgGGGaGaCtGGGAGCAGAGGGGGAT | chr9 | 12550058 | + | 4 |
| GCCTTCCTGCCGCCCTGTTTNNGRRT | GCCTTCCaGaCGCCCTGggTTCGAAT | chr9 | 21778500 | - | 4 |
| GAGGGCGCCAGGGAGCAGAGNNGRRT | GAGGGaGCaAGGGtGgAGAGATGGAT | chr9 | 52813698 | - | 4 |
| GCCTTCCTGCCGCCCTGTTTNNGRRT | GCCTTgCTGCCcCtCTGTTTGAGAAT | chr9 | 56745224 | - | 3 |
| GCCTTCCTGCCGCCCTGTTTNNGRRT | GgCTgCCTGCCtCCCTGcTTTGGGGT | chr9 | 57597270 | - | 4 |
| GAGGGCGCCAGGGAGCAGAGNNGRRT | GAGGGCGCCttGGtGCAGAGGGGAAT | chr9 | 58828390 | + | 3 |
| GAGGGCGCCAGGGAGCAGAGNNGRRT | GAGGGCcCCAGGGAGCAGAaATGGGT | chr9 | 61570158 | + | 2 |
| GAGGGCGCCAGGGAGCAGAGNNGRRT | GAGGGgGCgAGGGAGCAGgaAAGGAT | chr9 | 62050245 | + | 4 |
| GAGGGCGCCAGGGAGCAGAGNNGRRT | GAGGGaGggAGGGAGgAGAGATGGGT | chr9 | 62427411 | - | 4 |
| GAGGGCGCCAGGGAGCAGAGNNGRRT | cAGGcaGCCAGGtAGCAGAGCAGGGT | chr9 | 94444548 | + | 4 |
| GAGGGCGCCAGGGAGCAGAGNNGRRT | GgGGGgGCCAGGGAGtAGgGTGGGAT | chr9 | 98795872 | - | 4 |
| GGTCGTTGGTGGCAAAGAGTNNGRRT | GGTCaTTGGTGGaAtAGtGTCTGGAT | chr9 | 107894973 | + | 4 |
| GAGGGCGCCAGGGAGCAGAGNNGRRT | GAGGGCaCaAGGGAGCAGAGTGGAGT | chr9 | 121730051 | - | 2 |
| GAGGGCGCCAGGGAGCAGAGNNGRRT | GAGGGacCgtGGGAGCAGAGCTGAGT | chrX | 93787379 | - | 4 |
